# TGF-β1 potentiates Vγ9Vδ2 T cell adoptive immunotherapy of cancer

**DOI:** 10.1016/j.xcrm.2021.100473

**Published:** 2021-12-21

**Authors:** Richard E. Beatson, Ana C. Parente-Pereira, Leena Halim, Domenico Cozzetto, Caroline Hull, Lynsey M. Whilding, Olivier Martinez, Chelsea A. Taylor, Jana Obajdin, Kim Ngan Luu Hoang, Benjamin Draper, Ayesha Iqbal, Tom Hardiman, Tomasz Zabinski, Francis Man, Rafael T.M. de Rosales, Jinger Xie, Fred Aswad, Daniela Achkova, Chung-Yang Ricardo Joseph, Sara Ciprut, Antonella Adami, Helge G. Roider, Holger Hess-Stumpp, Balázs Győrffy, Jelmar Quist, Anita Grigoriadis, Anette Sommer, Andrew N.J. Tutt, David M. Davies, John Maher

**Affiliations:** 1 King’s College London, School of Cancer and Pharmaceutical Sciences, Guy’s Cancer Centre, Great Maze Pond, London SE1 9RT, UK; 2 Translational Bioinformatics, NIHR Biomedical Research Centre, Guy’s and St. Thomas’s NHS Foundation Trust and King’s College London, London SE1 9RT, UK; 3 Cancer Bioinformatics, King’s College London, School of Cancer and Pharmaceutical Sciences, Guy’s Cancer Centre, Great Maze Pond, London SE1 9RT, UK; 4 King’s College London, School of Biomedical Engineering and Imaging Sciences, St. Thomas’ Hospital, London SE1 7EH, UK; 5 Bayer Healthcare Innovation Center, Mission Bay, 455 Mission Bay Boulevard South, San Francisco, CA 94158, USA; 6 Bayer AG, Müllerstrasse 178, 13342 Berlin, Germany; 7 Department of Bioinformatics, Semmelweis University, Budapest H1085, Hungary; 8 Cancer Biomarker Research Group, Research Center for Natural Science, Budapest H1117, Hungary; 9 King’s College London, Breast Cancer Now Unit, School of Cancer and Pharmaceutical Sciences, Guy’s Cancer Centre, Great Maze Pond, London SE1 9RT, UK; 10 Department of Immunology, Eastbourne Hospital, Kings Drive, Eastbourne, East Sussex BN21 2UD, UK; 11 Department of Clinical Immunology and Allergy, King’s College Hospital NHS Foundation Trust, Denmark Hill, London SE5 9RS, UK; 12 Leucid Bio, Guy’s Hospital, Great Maze Pond, London SE1 9RT, UK

**Keywords:** Gamma delta T-cell, TGF-β, acute myeloid leukemia, prostaglandin E2, aminobisphosphonate, Ara-C

## Abstract

Despite its role in cancer surveillance, adoptive immunotherapy using γδ T cells has achieved limited efficacy. To enhance trafficking to bone marrow, circulating Vγ9Vδ2 T cells are expanded in serum-free medium containing TGF-β1 and IL-2 (γδ[T2] cells) or medium containing IL-2 alone (γδ[2] cells, as the control). Unexpectedly, the yield and viability of γδ[T2] cells are also increased by TGF-β1, when compared to γδ[2] controls. γδ[T2] cells are less differentiated and yet display increased cytolytic activity, cytokine release, and antitumor activity in several leukemic and solid tumor models. Efficacy is further enhanced by cancer cell sensitization using aminobisphosphonates or Ara-C. A number of contributory effects of TGF-β are described, including prostaglandin E_2_ receptor downmodulation, TGF-β insensitivity, and upregulated integrin activity. Biological relevance is supported by the identification of a favorable γδ[T2] signature in acute myeloid leukemia (AML). Given their enhanced therapeutic activity and compatibility with allogeneic use, γδ[T2] cells warrant evaluation in cancer immunotherapy.

## Highlights

• TGF-β enhances the yield and viability of expanded γδ T cells (γδ[T2] cells)
• γδ[T2] cells achieve more efficient tumor cell killing and cytokine release
• γδ[T2] cells traffic to bone marrow and resist suppression by TGF-β or PGE_2_
• γδ[T2] cells mediate enhanced antitumor and antileukemic activity

## Graphical abstract

**Figure:**
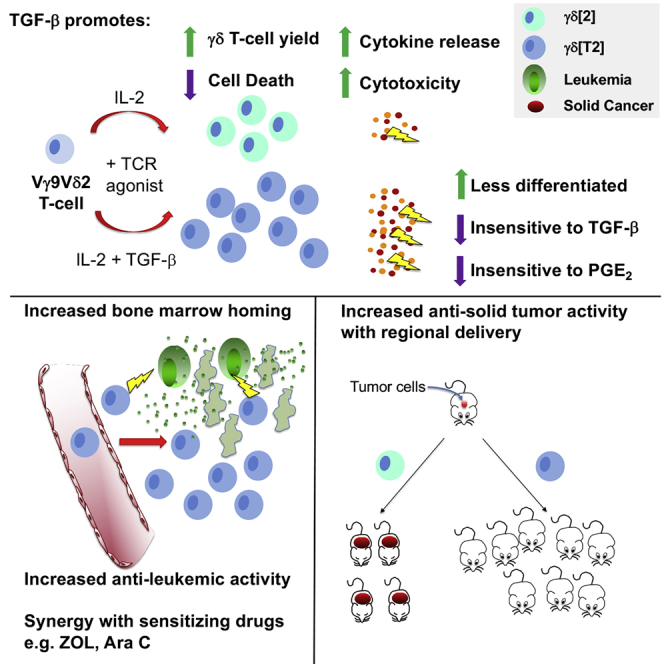

## Teaser

γδ T cells mediate immune surveillance and represent an attractive option for cancer immunotherapy. Here, Beatson et al. show that when circulating γδ T cells are expanded in the presence of TGF-β, they achieve enhanced bone marrow trafficking, tumoricidal activity, and cytokine release, enabling greater efficacy in leukemic and solid tumor models.

## Introduction

γδ T cells protect against diverse human cancers. Reconstitution of this minority T cell population following hematopoietic stem cell transplantation for leukemia is strongly linked to extended survival.^1^ Moreover, the presence of intratumoral Vγ9Vδ2 T cells is the most predictive leukocyte signature of improved outcomes across 25 cancers.^2^ These clinical observations reflect two attributes of γδ T cells. First, they undertake the human leukocyte antigen (HLA)-independent detection of metabolic, genomic, and signaling hallmarks of malignant transformation.^3^ The predominant human circulating γδ T cell population expresses a Vγ9Vδ2 T cell receptor (TCR) that recognizes mevalonate intermediates overproduced in tumor cells and known as phosphoantigens (PAg).^4^ γδ TCRs can also detect other tumor-associated antigens^5^ and stress ligands.^6^ A second key property of γδ T cells is their ability to coordinate both innate and adaptive immunity. Accordingly, γδ T cells can co-stimulate natural killer (NK) cells,^7^ promote dendritic cell maturation,^8^ and cross-present antigen to CD8^+^ αβ T cells.^9^

Circulating γδ T cells can be induced to proliferate using aminobisphosphonate drugs (e.g., zoledronic acid [ZOL]), which stimulate Vγ9Vδ2 T cells.^10^ Alternatively, antibody cross-linking of the γδ TCR induces the expansion of all γδ T cell subtypes.^11^ However, adoptive immunotherapy using γδ T cell products has only achieved a modest impact, mainly in hematological cancers.^12^ This highlights the need for strategies that more effectively harness the versatile antitumor activity of these cells.

Transforming growth factor (TGF) β is a pleiotropic cytokine that can confer regulatory properties on αβ and γδ T cells.^13^ Increasingly, however, it is evident that the effects of TGF-β are context dependent. An interleukin (IL)-17-producing phenotype is favored when IL-6, IL-1β, and IL-23 are also present.^14^ TGF-β can also promote the differentiation of CD103^+^ Vγ9Vδ2 T cells with enhanced cytolytic activity against E-cadherin-expressing solid tumor cells.^15^^,^^16^ Given the preeminent role of TGF-β in the tumor microenvironment,^17^ this intriguing finding raises the possibility that Vγ9Vδ2 T cells expanded in TGF-β could exploit a key node of tumor-associated immunosuppression for therapeutic benefit. However, the antitumor activity of TGF-β-conditioned Vγ9Vδ2 T cells remains untested. Here, we have evaluated this using a range of solid and leukemic cancer models, in addition to exploring the underling mechanisms and relevance to Vγ9Vδ2 T cells found within human cancers.

## Results

### Expansion of Vγ9Vδ2 T cells in serum-free medium containing TGF-β1 elicits a distinct immunophenotype with enhanced bone marrow migratory capacity

We hypothesized that the antileukemic activity of Vγ9Vδ2 T cells could be harnessed more effectively if their bone marrow trafficking capacity was amplified. CXCL12 recruits CXCR4-expressing cells to bone marrow,^18^ where endothelial E-selectin provides a portal of entry.^19^ Consequently, we set out to increase CXCR4 and E-selectin ligand expression by Vγ9Vδ2 T cells.

**Figure 1:**
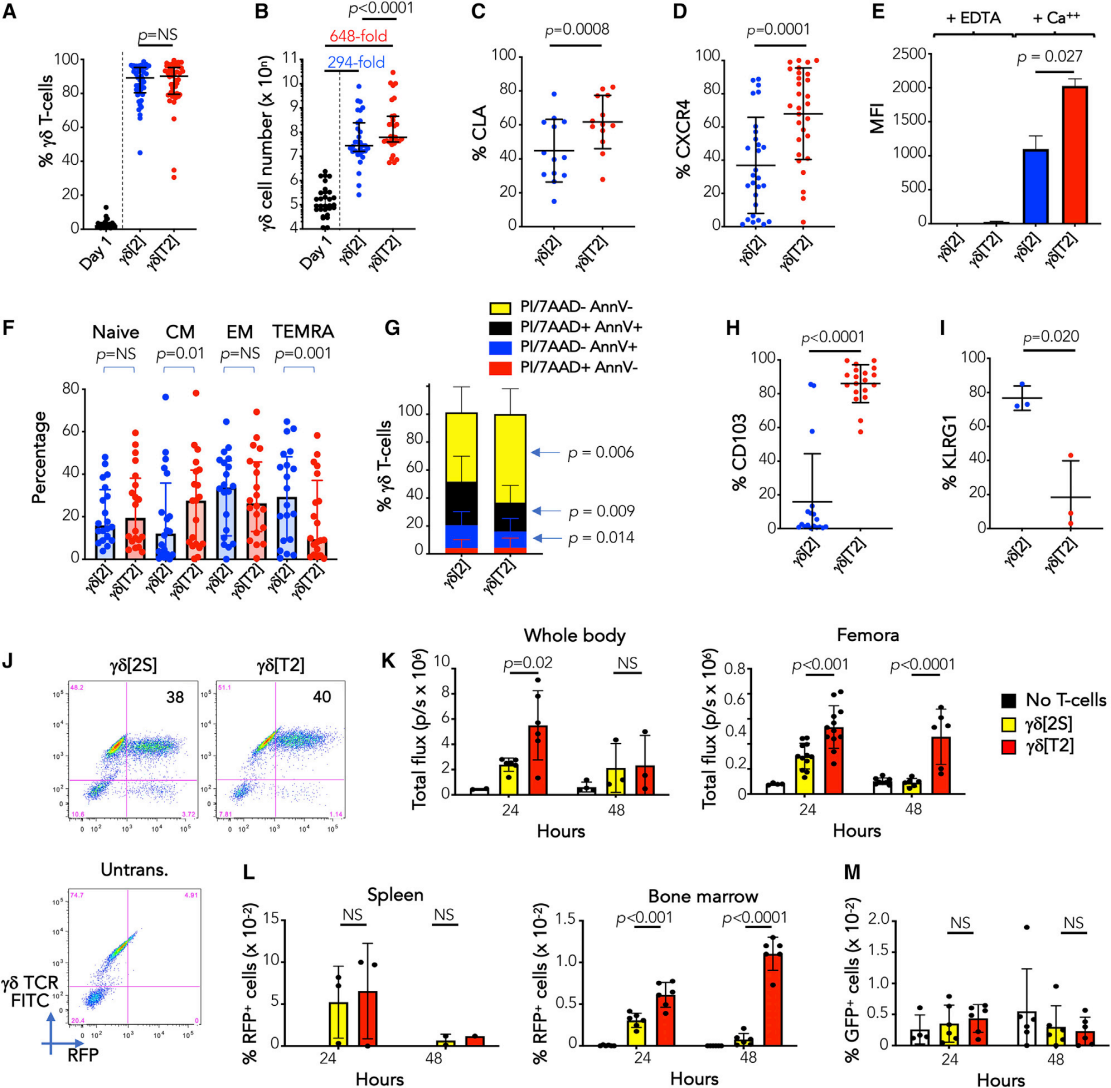
Expansion and characterization of γδ[T2] cells (A) Healthy donor PBMCs were activated with zoledronic acid (ZOL, n = 27) or immobilized anti-γδ TCR antibody (n = 15) and then cultured for 14–17 days in SFM containing IL-2 alone (γδ[2] cells) or IL-2 + TGF-β (γδ[T2] cells). The percentage of γδ T cells was measured in PBMCs (day 1) and after expansion in the indicated cytokines. (B) Absolute number of γδ T cells on day 1 and post-expansion. Median fold expansion is indicated for γδ[2] and γδ[T2] cells. (C–I) Post-γδ T cell expansion, the following markers were assessed in γδ[2] and γδ[T2] cells by flow cytometry: (C) % CLA; (D) % CXCR4; (E) binding to E-selectin-Fc fusion protein; (F) state of differentiation: naive (CD45RA^+^ CD27^+^), central memory (CM; CD45RA^−^ CD27^+^), effector memory (CD45RA^−^ CD27^−^), and terminally differentiated with CD45RA re-expression (TEMRA; CD45RA^+^ CD27^−^; (G) viability, apoptosis, and necrosis; (H) % CD103; and (I) % KLRG1. (J) γδ[T2] T cells and γδ T cells expanded from the same donor in human serum + IL-2 (γδ[2S]) were engineered to co-express firefly luciferase (ffLuc) and red fluorescent protein (RFP) and then analyzed by flow cytometry. GFP-expressing Jurkat cells were injected i.v. in NSG mice. After 4 days, 6 mice each were treated with 10 million RFP/ffLuc-expressing γδ[2S] or γδ[T2] T cells. Untrans., untransduced. (K) Mice were analyzed by BLI after 24 and 48 h to determine persistence of γδ T cells in the whole body and in a region of interest drawn around the femora (means ± SDs; n = 6 at 24 h; n = 3 at 48 h; 2-way ANOVA). (L) After each imaging session, 3 mice per group were culled. The % RFP^+^ (γδ) T cells present in spleen and bone marrow were determined (means ± SDs). (M) The % GFP^+^ (leukemic) cells present in bone marrow was also determined (means ± SDs). NS, not significant. (C)–(E) and (G)–(I) show means ± SDs and (A), (B), and (F) show medians ± interquartile ranges, in which data were or were not normally distributed, respectively. Accordingly, statistical analysis was performed using a Student’s t test or Wilcoxon signed-rank test, respectively.

When cultured in serum-free medium (SFM)^20^ or TGF-β1,^21^^,^^22^ αβ T cells upregulate E-selectin ligands (e.g., cutaneous lymphocyte antigen [CLA]) and CXCR4.^23^ To test whether Vγ9Vδ2 T cells respond similarly, peripheral blood mononuclear cells (PBMCs) were activated with ZOL, or anti-pan γδ TCR, and Vγ9Vδ2 T cells were expanded in SFM containing IL-2 alone (γδ[2] cells) or IL-2 + TGF-β (γδ[T2] cells). In both cases, gd T cells enriched to equivalent purity (Figure 1A). As expected, a greater proportion of non-Vδ2 cells were present when antibody cross-linking rather than ZOL was used (Figure S1A). We also found that Vγ9Vδ2 T cell yield was significantly greater when TGF-β was added (Figure 1B). While we noted considerable donor-to-donor heterogeneity, CLA (Figure 1C), CXCR4 (Figure 1D), and calcium-dependent E-selectin binding activity (Figure 1E) were significantly greater in γδ[T2] cells. TGF-β also retarded differentiation (Figure 1F), with increased CCR7 and CD27 and reduced CD45RA expression (Figure S1B). γδ[T2] cells were more activated, indicated by elevated CD25 and CD69 and reduced CD62L (Figure S1C). Moreover, baseline apoptosis, necrosis (Figure 1G), and camptothecin-induced death (Figure S1D) were reduced in γδ[T2] cells, accompanied by significantly increased expression of anti-apoptotic molecules (e.g., cIAP-1, XIAP) and a reduction in pro-apoptotic molecules (e.g., DR5, FADD) (Figure S1E) and cytokines (e.g., tumor necrosis factor α [TNF-α], which promotes γδ T cell apoptosis^24^; Figure S1F). Given that TGF-β has been shown to inhibit Vγ9Vδ2 T cell proliferation,^16^ we believe that the reduction in cell death accounts for the enhanced yield of γδ [T2] cells. Further phenocopying the effect of TGF-β on αβ T cells,^25^ CD103 (α_E_ integrin) was markedly upregulated on γδ[T2] cells (Figure 1H). Expression of the alternative E-cadherin ligand and late-stage differentiation/exhaustion marker KLRG1 was proportionately reduced (Figure 1I). Although FoxP3 was detectable in γδ[2] and γδ[T2] cells, the levels were lower than in CD4^+^ regulatory T cells (Treg; Figures S1G and S1H). Moreover, neither γδ[T2] and γδ[2] cells suppressed the proliferation of activated CD4^+^ T cells (Figure S1I). Production of IL-10, a cytokine produced by some regulatory Vγ9Vδ2 T cells,^26^ was also significantly lower by γδ[T2] cells (Figure S1J). We were unable to address whether TGF-β exerted these effects directly since the expansion of purified Vγ9Vδ2 T cells in SFM was not robust in our hands. Our data suggest that γδ[T2] cells are more closely related to TGF-β-induced CD103^+^ cytotoxic Vγ9Vδ2 T cells^15^ than are Vγ9Vδ2 Tregs.^13^

We next evaluated the bio-distribution of infused γδ[T2] cells *in vivo*. Studies were undertaken in mice since interactions involving CXCR4^27^ and E-selectin^28^ both cross the human/mouse species barrier. Human γδ[T2] cells were engineered to co-express firefly luciferase (ffLuc) and red fluorescent protein (RFP; Figure 1J) and were injected intravenously (i.v.) into NSG mice in which Jurkat-GFP leukemia had been established. To determine the combined effect of SFM and TGF-β, comparison was made with Vγ9Vδ2 T cells that had been expanded in serum-containing medium with IL-2 alone (γδ[2S] cells; Figure 1J). Using bioluminescence imaging (BLI; Figure 1K) and flow cytometry (Figure 1L), a significant increase in the migration of γδ[T2] cells to bone marrow/femora, but not spleen, was seen at 24 and 48 h. At these early time points, antileukemic activity was not evident (Figure 1M). Orthogonal demonstration of bone marrow entry was obtained by positron emission tomography/computed tomography (PET/CT) imaging of [^89^Zr]Zr(oxinate)_4_-labeled γδ[T2] cells.^29^ Signal was clearly visualized in both femora and vertebrae (Figures S2A and S2B), with confirmation by bio-distribution analysis (Figure S2C).

### γδ[T2] cells demonstrate enhanced *in vitro* antileukemic activity

**Figure 2:**
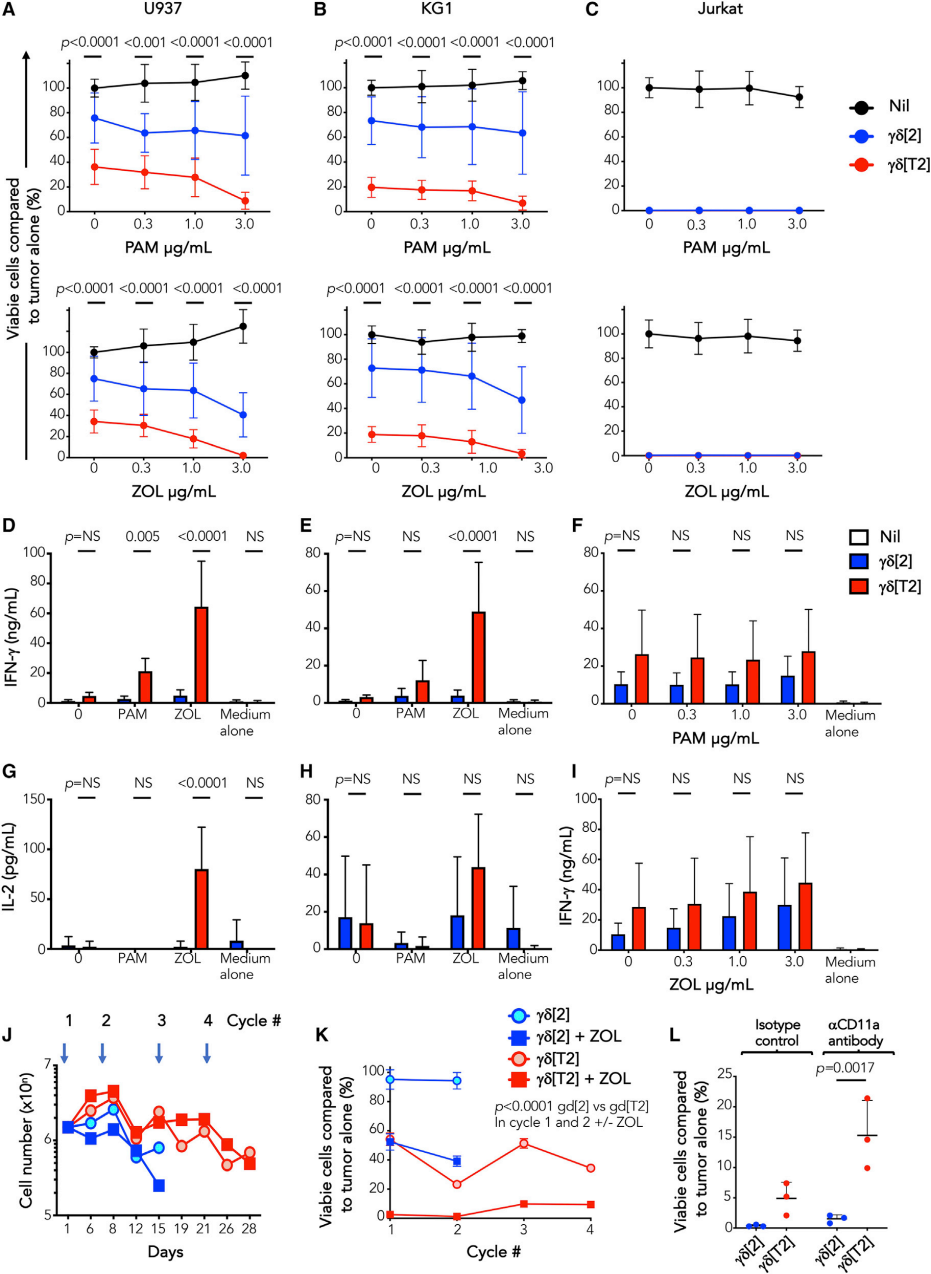
*In vitro* antileukemic activity of γδ[T2] cells (A–C) γδ[2] and γδ[T2] cells were expanded from healthy donors and co-cultivated at a 5:1 E:T ratio with ffLuc^+^ leukemic cell lines. Where indicated, leukemic cells were pre-sensitized by incubation for 24 h with the indicated concentration of ZOL or PAM before the addition of γδ T cells. After a further 24 h, leukemic cell viability was measured by luciferase assay. Data are shown for (A) U937, (B) KG-1, and (C) Jurkat cells. Note the absence of residual viable cells in Jurkat/γδ T cell co-cultures. The p values shown above each E:T ratio in (A) and (B) compare cytotoxicity by γδ[2] versus γδ[T2] cells. (D–I) The following cytokines were measured by ELISA in supernatants harvested from co-cultures described above after 24 h (n = 3–6; mean ± SD). (D) IFN-γ-U937 cells, (E) IFN-γ-KG1 cells, (F) IFN-γ-Jurkat cells (±PAM sensitization), (G) IL-2-U937 cells, (H) IL-2-KG1 cells, (I) IFN-γ-Jurkat cells (±ZOL sensitization). (J and K) γδ[2] and γδ[T2] cells were serially re-stimulated by addition of ffLuc^+^ U937 cells without exogenous cytokine (1:1 E:T ratio; timing indicated by overhead arrows). Where indicated, leukemic cells were pre-sensitized for 24 h using ZOL (3 μg/mL). (J) Number of γδ T cells over time is indicated (mean, n = 3). (K) Residual viability of leukemic cells at 24 h was determined by luciferase assay (means ± SDs, n = 3; 2-way ANOVA). Data are representative of 3 independent replicates that showed similar findings. (L) KG-1 cells were sensitized with ZOL (1 μg/mL) for 24 h before addition of γδ[2] or γδ[T2] cells (E:T ratio 5:1), together with a CD11a-blocking antibody or isotype control. Viable KG1 cells (%) that remained post co-culture are plotted (means ± SDs, n = 3 independent donors; 1-way ANOVA).

We next evaluated the efficacy of γδ[T2] cells in leukemic models. Owing to the ease of production, ZOL-activated cells were used. Greater killing of U937 and KG-1 cells was mediated by γδ[T2] cells compared to γδ[2] cells (Figures 2A and 2B), potentiated by target pre-exposure to pamidronate (PAM) or ZOL.^30^ Jurkat cells proved highly sensitive to killing by both γδ[T2] and γδ[2] cells (Figure 2C). Activated γδ[T2] cells released more interferon γ (IFN-γ) and IL-2 (Figures 2D–2I) and maintained cytotoxic function upon repeated leukemic cell addition (Figures 2J and 2K), unlike γδ[2] cells.

**Figure 3:**
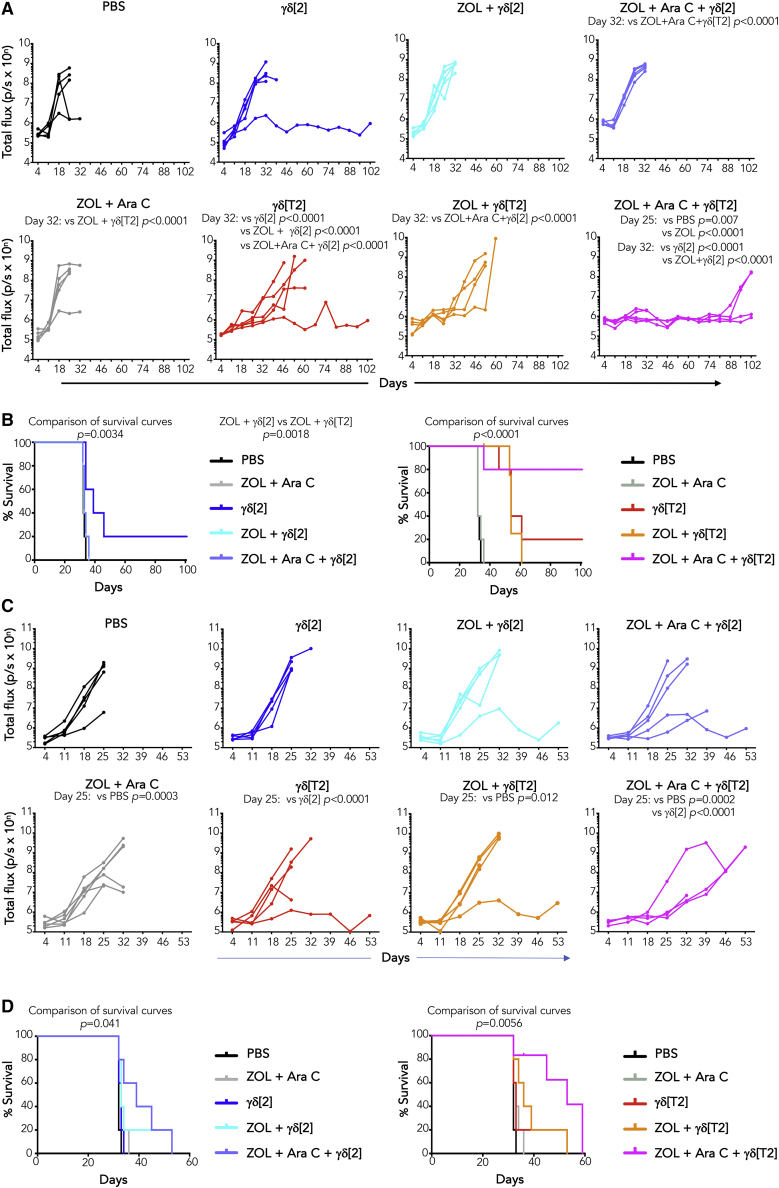
Immunotherapy of leukemia with γδ[T2] cells (A) NSG mice were inoculated i.v. with 1 × 10^5^ ffLuc^+^ Jurkat cells on day 1. Indicated groups of mice received ZOL (20 μg i.v.) on day 5; Ara-C (110 mg/kg i.v.) on day 5, and/or γδ[2] or γδ[T2] T cells (4 × 10^6^ cells) i.v. on day 6. Serial BLI emission from individual mice is shown (2-way ANOVA). (B) Kaplan-Meier survival curves of mice shown in (A) (log-rank [Mantel-Cox] test). Mice treated with γδ[2] (left) or γδ[T2] T cells (right) are shown in separate panels for clarity of presentation. One mouse in the ZOL + Ara-C + γδ[T2] group died of infection while leukemia free. (C) NSG mice were inoculated i.v. with 1 × 10^5^ ffLuc^+^ KG1 cells on day 1. Indicated groups of mice received ZOL 20 μg i.v. on day 5; 110 mg/kg Ara-C i.v. on day 5, and/or γδ[2] or γδ[T2] T cells (4 × 10^6^ cells) i.v. on day 6. Serial BLI of individual mice is shown (2-way ANOVA). (D) Kaplan-Meier survival curves of mice shown in (C) (log-rank [Mantel-Cox] test). Mice treated with γδ[2] (left) or γδ[T2] T cells (right) are shown in separate panels.

Many cytotoxic drugs sensitize tumor cells to killing by γδ T cells.^31^^,^^32^ We observed that sublethal exposure to the antileukemic drug, Ara-C (Figure S3A) rendered both U937 and KG1 cells more susceptible to killing by γδ[T2] T cells, with further enhancement by ZOL (Figures S3B and S3C). In contrast to PAM or ZOL, sensitization by Ara-C did not increase IFN-γ release (Figures S3D and S3E) or upregulate NKG2D ligands on leukemic cells (Figures 3F and 3G). However, cleaved caspase 3 levels in U937 and KG1 cells were increased, suggesting that Ara-C lowered the threshold for Vγ9Vδ2 T cell-mediated killing (Figures S3H and S3I).

### γδ[T2] cells mediate enhanced *in vivo* anti-leukemic activity

*In vivo* antileukemic activity was first tested in a Jurkat xenograft model. Treatment with i.v. γδ[T2] cells alone delayed disease progression, compared to γδ[2] cells (Figure 3A). While pre-treatment with ZOL had a marginal effect, the combination of ZOL + Ara-C boosted response to γδ[T2] cells, improving both disease control (Figure 3A) and survival (Figure 3B). By contrast, neither ZOL + Ara-C alone or combined with γδ[2] cells was effective (Figures 3A and 3B). We next evaluated sensitization by Ara-C alone and found that this also potentiated the efficacy of γδ[T2] cells against Jurkat leukemia (Figure S4A), leading to prolonged survival (Figure S4B).

To test this in a more challenging setting, we selected the KG1 leukemia model. Despite *in vitro* sensitivity, KG1 xenografts are poorly responsive to γδ[T2] or γδ[2] cells (Figure 3C). Nonetheless, when mice were sensitized with ZOL + Ara-C, γδ[T2] cells delayed disease progression (Figure 3C) and enhanced survival (Figure 3D). γδ[T2] cells also delayed disease progression (Figure S4C) and prolonged the survival of mice with an established U937 xenograft (Figure S4D).

### γδ[T2] cells undergo enhanced activation against solid tumor cells but require regional delivery for efficacy

**Figure 4:**
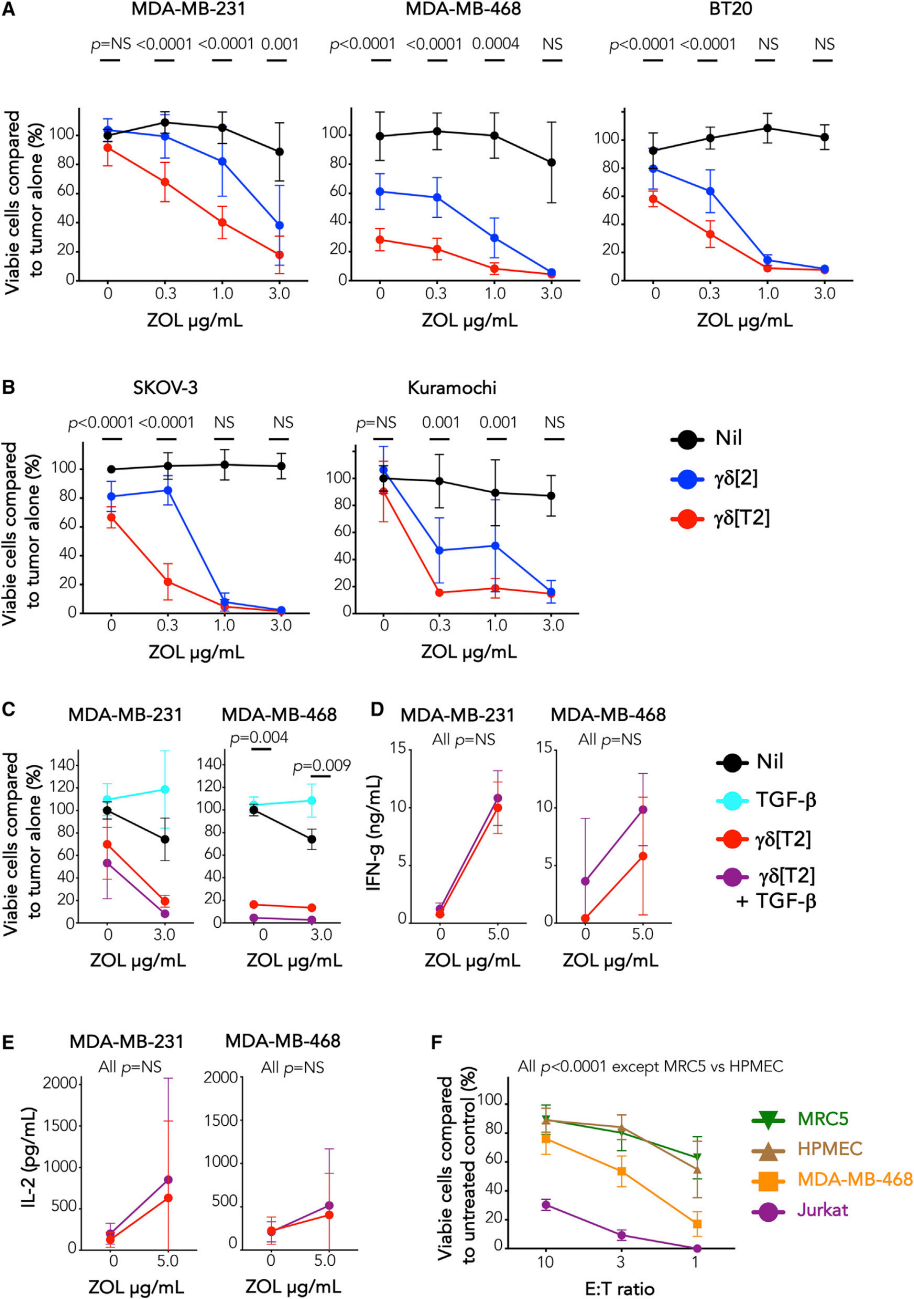
*In vitro* antitumor activity of γδ[T2] cells (A) 2 ×10^4^ of the indicated TNBC cell lines were pulsed with ZOL for 24 h, followed by co-culture with γδ[2] and γδ[T2] cells (E:T ratio 5:1). After 24 h, tumor viability was measured by luciferase or MTT assay. p values compare cytotoxicity by γδ[2] and γδ[T2] cells at each E:T ratio (2-way ANOVA). (B) 2 ×10^4^ of the indicated ovarian cancer cell lines were pulsed with ZOL for 24 h, followed by co-culture with γδ[2] and γδ[T2] cells (E:T ratio 5:1). After 24 h, tumor viability was measured by luciferase or MTT assay. p values compare cytotoxicity by γδ[2] and γδ[T2] cells at each E:T ratio (2-way ANOVA). (C) Evaluation of cytotoxic activity of γδ[T2] T cells against TNBC cells by luciferase assay ± exogenous TGF-β (2-way ANOVA, comparing γδ[T2] versus γδ[T2] + TGF-β). Where indicated, monolayers were pulsed for 24 h with ZOL before the addition of γδ T cells for a further 24 h. (D and E) Before cytotoxicity assays, supernatants were collected from co-cultures in (C) and analyzed for (D) IFN-γ (means ± SDs, n = 3; 2-way ANOVA) and (E) IL-2 (means ± SDs, n = 3; 2-way ANOVA). p values in (C)–(E) compare cytotoxicity and cytokine release by γδ[T2] cells versus γδ[T2] cells + TGF-β. (F) γδ[T2] cells were co-cultivated for 24 h at the indicated E:T ratio with transformed (Jurkat, MDA-MB-468) or non-transformed human pulmonary endothelial cells (HMPEC) or fibroblasts (MRC5 cells). Residual target cell viability was determined by MTT or luciferase assay (means ± SDs, 9–12 replicates from 3–4 independent experiments). p values compare γδ[T2]-mediated killing of any target cell pair across all E:T ratios (2-way ANOVA, comparing any pair of target cells, except MRC5 and HMPEC).

Given the enhanced antitumor activity of γδ[T2] cells in models of hematological malignancy, we wondered whether enhanced function would also be seen in solid tumor models. When compared to γδ[2] cells, γδ[T2] cells enhanced cytotoxicity against triple negative breast cancer (TNBC; Figures 4A and S5A) and ovarian cancer cells (Figure 4B), accompanied by the increased release of several cytokines and chemokines (Figure S5B). Notably, the production of pro-tumorigenic IL-17 was negligible (Figure S5B).^33^ Neither cytotoxicity (Figure 4C) nor cytokine release (Figures 4D and 4E) by γδ[T2] cells was suppressed by exogenous TGF-β1. Moreover, γδ[T2] cells demonstrated preferential cytotoxic activity against transformed compared to non-transformed cell types (Figure 4F).

To test *in vivo* function, mice with established orthotopic MDA-MB-231 tumors were treated with ZOL followed by i.v. γδ[2] or γδ[T2] cells after 24 h and IL-2 at 72 h. However, no therapeutic activity was elicited by either Vγ9Vδ2 T cell population *in vivo* (Figure S5C). Given their altered trafficking properties, we hypothesized that γδ[T2] cells had preferentially migrated to bone marrow rather than to tumor. To investigate this, we studied the trafficking of ffLuc/RFP-engineered γδ[T2] cells after i.v. delivery to tumor-bearing mice. Using BLI, we observed the migration of infused cells to femora (Figure S5D). Moreover, RFP^+^ cells were retrieved from bone marrow but not tumor at 48 h (Figure S5E), indicating that influx at the site of disease was inadequate. These data also reaffirm the potential utility of γδ[T2] cell immunotherapy in the treatment of bone marrow malignancy.

**Figure 5:**
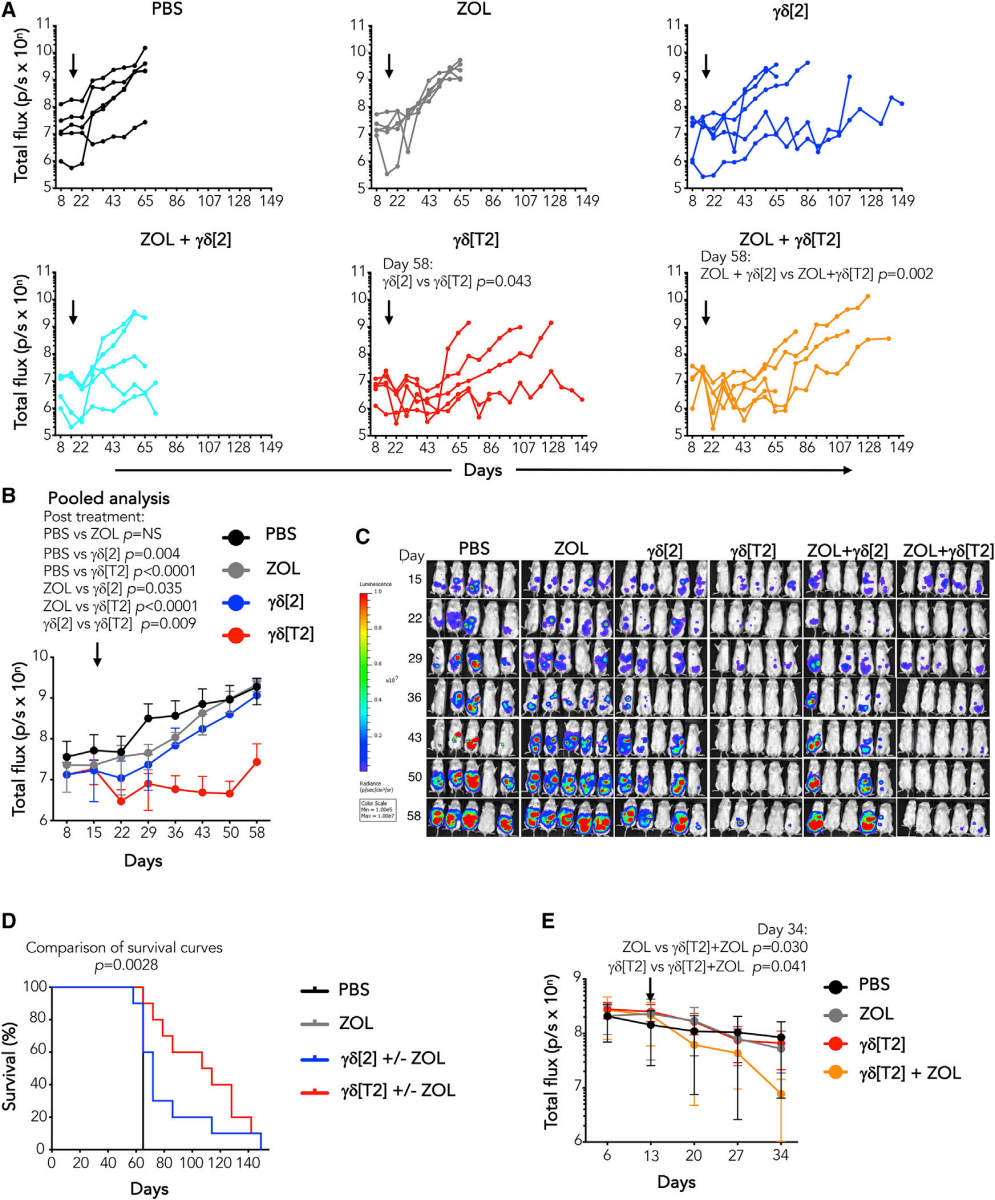
Immunotherapy of solid tumors with regional delivery of γδ[T2] cells (A) Firefly luciferase-expressing SKOV-3 ovarian tumor xenografts were established for 15 days in NSG mice. Mice were treated with i.p. ZOL (20 μg) or PBS. After 24 h, mice received 2.5 × 10^6^ γδ[2] or γδ[T2] cells i.p. Serial BLI emission from mice is shown (2-way ANOVA). (B) Means ± SDs of BLI emission from groups is shown. Since ZOL did not influence therapeutic outcome, data from γδ[2] ± ZOL groups and γδ[T2] ± ZOL groups have been pooled (2-way ANOVA). (C) BLI images of mice using the same scale throughout the experiment are presented. (D) Kaplan-Meier analysis of data shown in (A)–(C) (log-rank [Mantel-Cox] test). (E) NSG mice were inoculated with ffluc^+^ Kuramochi tumor cells. After 13 days, mice were allocated to treatment groups with similar average tumor burden for i.p. treatment with 40 μg ZOL or PBS as control. After 24 h, indicated mice received 1.5 × 10^6^ γδ[T2] T cells i.p. Tumor status was monitored by serial BLI (2-tailed Student’s t test).

To circumvent the need for γδ[T2] cell migration to solid tumors, we tested whether intraperitoneal (i.p.) delivery may prove useful in ovarian cancer treatment, given its tendency for loco-regional rather than distant spread. In NSG mice with established i.p. ffLuc SKOV-3 tumors, treatment with γδ[T2] cells alone or with ZOL resulted in improved disease control (Figures 5A–5C) and survival (Figure 5D), compared to γδ[2] cells. To test this in a second system, xenografts were established using ffLuc^+^ Kuramochi cells, which represents the most representative cell line model of high-grade serous ovarian cancer.^34^ Tumors were unstable, poorly vascularized, and partially sensitive to ZOL (Figure 5E). Nonetheless, co-treatment with γδ[T2] cells led to a further reduction in disease burden (Figure 5E).

### Mechanistic investigation of γδ[T2] cells

**Figure 6:**
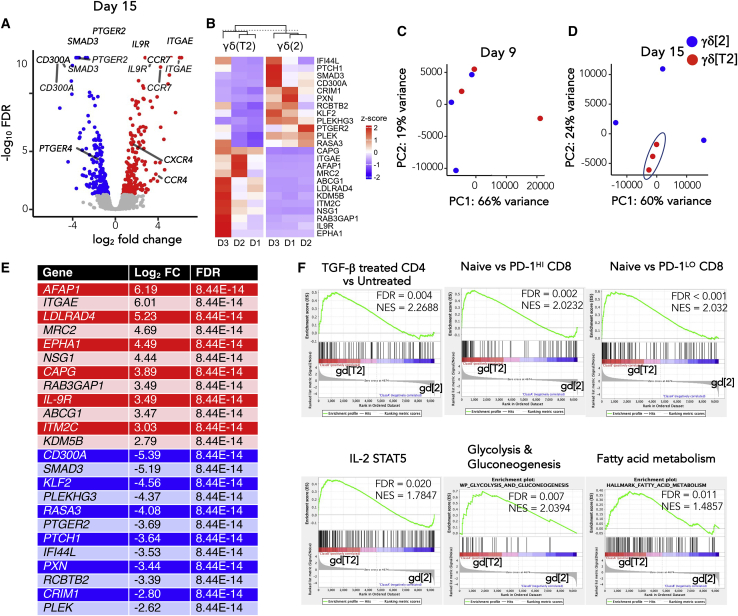
Differentially expressed genes in γδ[2] versus γδ[T2] cells (A) RNA-seq analysis was performed on *ex vivo* expanded γδ[2] and γδ[T2] cells from 3 healthy donors, purified on day 15 of culture by flow sorting. Volcano plot of differential expression analysis results, with genes of interest labeled. (B) A heatmap shows donor-to-donor variability of expression of top differentially expressed genes in γδ[2] and γδ[T2] cells across 3 independent donors (D1–D3). (C and D) Principal-component analysis plot of transcripts on day 9 (C) and (D) on day 15 following purification of γδ T cells by flow sorting. (E) Most significantly differentially expressed genes identified in day 15 samples based on FDR and corresponding log_2_ FC. Upregulated genes in γδ[T2] cells compared to γδ[2] cells are shown in the red section, while downregulated genes (indicated by −log_2_ FC values) are listed in the blue section. (F) Gene set enrichment analysis was performed on normalized expression data (transcripts per million). Enrichment plots of genes associated with TGF-β signaling, naive phenotype, IL-2/STAT5 signaling, glycolysis and gluconeogenesis, and fatty acid metabolism are shown in γδ[T2] compared to γδ[2] cells. FC, fold change; FDR, false discovery rate; NES, normalized enrichment score.

Bulk RNA sequencing (RNA-seq) was performed on flow sorted γδ[T2] and γδ[2] cells on days 9 and 15 of culture (n = 3 independent donors; Tables S1 and S2) to identify differentially expressed genes and pathways between these cells. A total of 55 genes (fold change [FC] ≥4 or ≤0.25 and false discovery rate [FDR] < 0.01) were apparent on day 9 (Table S1), 29 of which were unique to this time, while 26 were still present on day 15. On day 15, 109 genes were noted, 82 of which were unique to this time (Figure 6A). TGF-β1-responsive genes (e.g., *ITGAE*, *LDLRAD4*, *MRC2*, *SMAD3*) were modulated in the expected manner at both time points, confirming the validity of this approach. Nevertheless, there was considerable donor-to-donor variability in gene expression, consistent with the heterogeneity of these *ex vivo* expanded Vγ9Vδ2 T cell cultures (Figure 6B).

Principal-component analysis (PCA) of the expression profiles at day 9 showed substantial similarity between γδ[2] and γδ[T2] samples (Figure 6C), suggesting that phenotypic effects of TGF-β were not mature by this time. By contrast, γδ[T2] samples exhibited clear clustering and separation from γδ[2] samples by day 15 (Figure 6D). Given these findings, we advanced the day 15 RNA-seq dataset for further analysis. The top 24 differentially expressed genes on day 15 were designated “γδ[T2] cell signature” (Figure 6E) and included upregulation of *ITGAE* (α_E_ integrin; CD103), *IL9*, and *IL9R*, as noted previously.^16^ Despite differences in culture methods and RNA analysis methodology, when we compared the 229 genes with FDR < 0.05 in the Peters dataset and our dataset, the Spearman correlation between the log FCs was found to be 0.81 (p < 2.2^e−16^), emphasizing the strong similarity of these 2 cell populations. We also observed significantly elevated *CCR4*, *CCR7*, and *CXCR4* expression in γδ[T2] cells. Several inhibitory receptors were downregulated, notably prostaglandin (PG)E_2_ receptors (*PTGER2* and *PTGER4*), *CD300A*, and *KLRG1* (Figures 6A and 6B; Tables S1 and S2). Using gene set enrichment analysis (GSEA), we identified the overrepresentation of genes associated with TGF-β signaling, naive phenotype, IL-2/STAT5 signaling, glycolysis and gluconeogenesis, and fatty acid metabolism in γδ[T2] cells, compared to γδ[2] cells (Figure 6F; Table S3).

**Figure 7:**
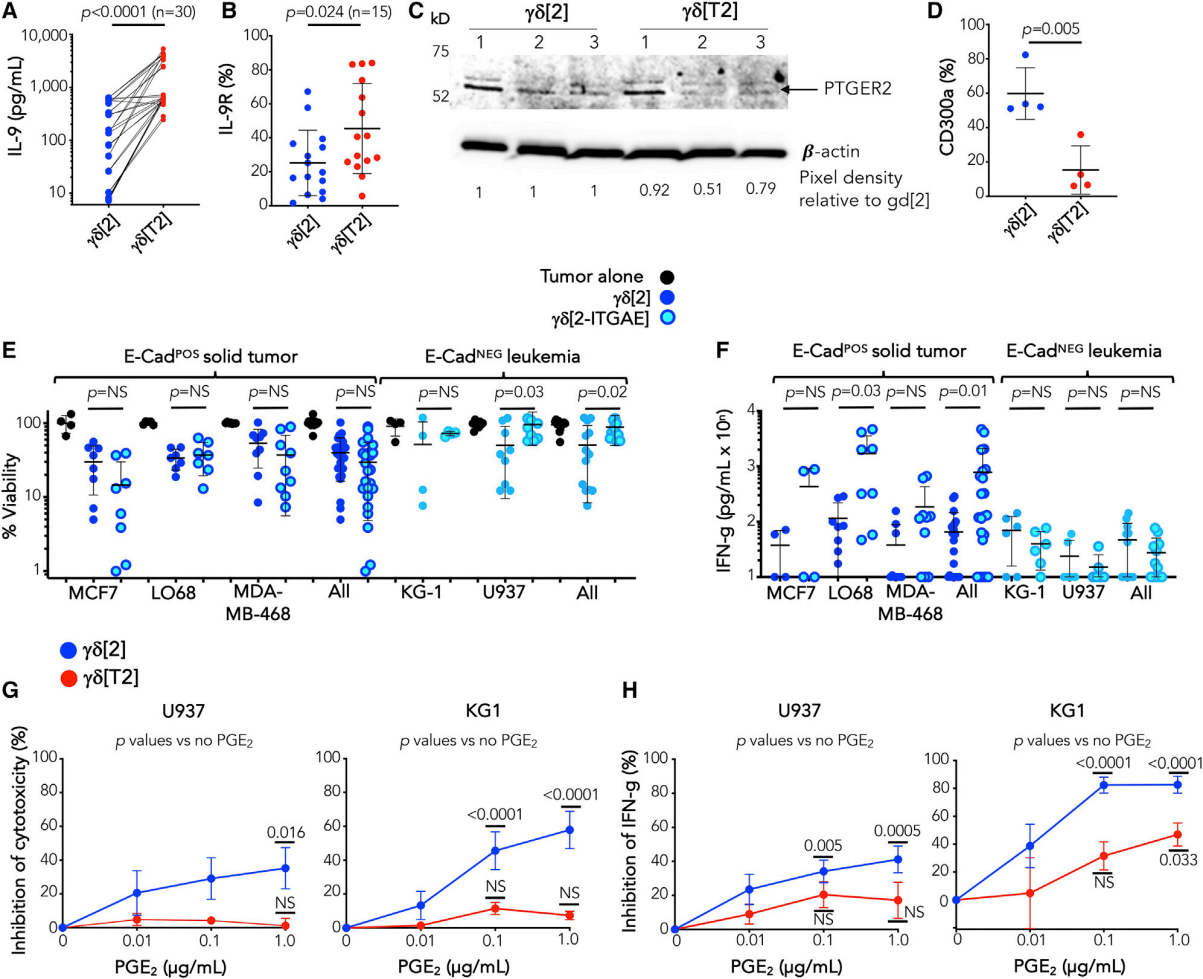
Functional investigation of differentially expressed genes in γδ[2] versus γδ[T2] cells (A) IL-9 was measured in supernatants harvested on day 15 from γδ[2] and γδ[T2] cells (values for individual donors are linked by a line; unpaired 2-tailed Student’s t test). (B) IL-9 receptor expression was quantified on γδ[2] and γδ[T2] cells on day 15 using flow cytometry (unpaired 2-tailed Student’s t test). (C) PTGER2 protein expression in γδ[2] and γδ[T2] cells (n = 3 independent donors) was determined by western blotting under reducing conditions. Quantification of band pixel density was performed using ImageJ. Data were normalized for each γδ[T2] cell sample with respect to γδ[2] cells expanded from the same donor. (D) CD300a was quantified on day 15 from γδ[2] and γδ[T2] cells using flow cytometry (unpaired Student’s t test). (E) The indicated ffLuc-expressing tumor cell lines were co-cultivated with γδ[2] and γδ[2-ITGAE] cells for 24 h at 1:1 E:T ratio. Tumor cell viability is shown in comparison with tumor cells that had been cultured alone (means ± SDs; 1-way ANOVA). (F) Supernatants were collected from co-cultures described in (E) after 72 h and analyzed for IFN-γ (means ± SDs; unpaired Student’s t test). (G) Effect of exogenous PGE_2_ on cytotoxicity (means ± SEMs, n = 10 from 5 independent donors) by γδ[2] and γδ[T2] cells when co-cultivated with U937 and KG-1 cell lines for 72 h at 1:1 E:T ratio. Data were normalized with respect to cytotoxicity by γδ[2] and γδ[T2] cells in the absence of added PGE_2_ (2-way ANOVA). NS, not significant. (H) Effect of exogenous PGE_2_ on IFN-γ production (means ± SEMs, n = 8 from 4 independent donors) by co-cultures described in (G). Data were normalized with respect to IFN-γ production by γδ[2] and γδ[T2] cells in the absence of added PGE_2_ (2-way ANOVA). “All” in (E) and (F) refers to pooled data from solid tumor and leukemic cell lines, respectively.

Many of these transcriptional changes were validated at the protein level. IL-9 was produced at variable levels by γδ[2] cells, and this was consistently increased in γδ[T2] cells (Figure 7A), as was IL-9 receptor expression (Figure 7B). Despite the considerable donor-to-donor variability, IL-9 production by γδ[2] and γδ[T2] cells were strongly correlated (Spearman *r* = 0.475, p = 0.0092), suggesting that some donors have a greater intrinsic capacity to generate IL-9-producing γδ[T2] cells. PTGER2 was identified by immunoblotting as a 53-kDa doublet, as described.^35^ Levels were variably reduced in γδ[T2] compared to γδ[2] cells obtained from 3 different donors (Figure 7C), while cell surface CD300a expression was also significantly reduced in γδ[T2] cells (Figure 7D).

Three candidates were prioritized for further investigation, namely IL-9, CD103, and PTGER2. Antibody-mediated blockade of IL-9 during the expansion of γδ[T2] cells had a negligible effect on their cytotoxic activity or activation-induced IFN-γ release (Figures S6A and S6B). Conversely, the addition of IL-9 during the expansion of γδ[2] cells also failed to alter these parameters (Figures S6C and S6D), arguing against an important role of this autocrine loop in the *in vitro* phenotypic effects of TGF-β. While antibody-blocking experiments targeted against CD103 proved inconclusive, retroviral expression of CD103 expressed in γδ[2] cells (γδ[2-ITGAE] cells) promoted a trend toward enhanced cytotoxicity (Figure 7E) and IFN-γ release (Figure 7F) in co-cultures with E-cadherin^+^ solid tumor cells. These data are consistent with a prior study of TGF-β-conditioned Vγ9Vδ2 T cells.^15^ Importantly, however, a trend in the opposite direction was observed when γδ[2-ITGAE] cells were co-cultured with leukemic cells that lack E-cadherin (Figures 7E and 7F). These data do not support a role for CD103 in the enhanced antileukemic activity of γδ[T2] cells. Since the expression of both *PTGER2* and *PTGER4* was reduced in γδ[T2] cells, we next investigated their sensitivity to suppression by PGE_2_. We focused on hematological models given the lack of involvement of CD103 or IL-9 in the enhanced antileukemic activity of γδ[T2] cells and the reported production of PGE_2_ by leukemic blasts.^36^ When PGE_2_ was added to leukemic cell co-cultures, γδ[2] cell cytotoxicity and IFN-γ release were inhibited in a dose-dependent manner. By contrast, there was significantly less impact on γδ[T2] cell function (Figures 7G and 7H). Given the role of CD103 in the killing of solid tumor cells, we next assessed the role of another integrin, LFA-1, in leukemic cell killing. We found that anti-CD11a neutralizing antibodies significantly inhibited the killing of KG-1 targets by γδ[T2] but not γδ[2] cells (Figure 2L), implicating enhanced LFA-1 activity in this process.

### A γδ[T2] cell signature is associated with improved prognosis in acute myeloid leukemia (AML)

Finally, we used bioinformatic approaches to explore whether γδ[T2]-like cells are present in human cancer and influence disease outcomes. Using the Cancer Genome Atlas Genomic Data Commons (TCGA GDC) datasets, we found that *TRDV2* transcripts were detected in all 22 cancers evaluated (Figure S7A**,** top panel), with the highest expression in AML (n = 128 leukemias; Figure S7A, center panel). Moreover, there was a clear survival benefit for AML patients when high *TRDV2* and *IL9* expression coincided (Figure S6E). As previously reported,^2^ *TRDV2* expression was associated with improved prognosis across all cancers (n = 8,369 cancers; Figure S7B).

Given the disproportionately high percentage of *TRDV2*^+^ AMLs and thymomas, we next assessed the prognostic association of transcripts encoding TCR subunits found in Vγ9Vδ2 cells, making comparisons with Vδ1 and αβ TCR subunits. Only high levels of *TRDV2* and *TRGV9* transcripts were associated with significantly improved survival in AML. By contrast, all five transcripts showed a positive survival association in thymoma, with three reaching significance (Figure S7C).

Next, we tested whether the 24 gene γδ[T2] cell signature described above (Figure 6E) was specifically associated with Vγ9Vδ2 cells in AML and thymoma. A correlation analysis was performed between the *Z* scores for each of our 24 signature genes and the correlation coefficient for each signature gene and the relevant TCR transcript, derived from TCGA datasets (Figure S7D). By this means, we set out to ascertain whether the 24 genes that comprise the γδ[T2] cell signature were similarly associated (in terms of direction and strength) with either αβ or γδ TCR genes, both in our *in vitro* cultured cells and those hypothesized to be present in AML or thymoma. More important, we found significant positive associations between the γδ[T2] cell signature and both *TRGV9* and *TRDV2* in AML, but not thymoma. No such associations were seen in either disease with *TDRV1*, *TRAC*, or *TRBC2*.

These findings support the hypothesis that the phenotype of Vγ9Vδ2 T cells found in AML resembles that of γδ[T2] cells. To further test this, we assessed *TGFB1* transcripts in all 22 cancers indicated above and found that levels were significantly higher in AML than all other tumors (Figure S7A, lower panel). The abundant co-expression of *TRDV2* and *TGFB1* transcripts in AML suggests that a suitable environment may be present in this cancer to favor TGF-β conditioning of Vγ9Vδ2 T cells. Next, we examined whether the γδ[T2] cell signature had prognostic significance across a broader panel of 38 cancers. We assessed the impact of each individual gene using PRECOG (prediction of clinical outcomes from genomic profiles).^2^ This revealed a strong association between the γδ[T2] cell signature and positive outcome in AML and neuroblastoma, while a weaker association was found in B cell acute lymphoblastic leukemia (Figure S7E). In keeping with this, γδ[T2] cells not only elicited superior antitumor activity in myeloid leukemic models (see above) but also were significantly more cytolytic against the SH-SY5Y neuroblastoma cell line (Figure S5F). Next, we assessed the prognostic association of the combined 24 gene γδ[T2] cell signature. Higher expression was linked to longer overall survival in AML, but only if leukemias were positive for *TRDV2* expression (Figure S7F). A similar trend was seen in thymoma and also the remaining 20 tumors in which lower expression of *TRDV2* was present (Figure S7F). Finally, we assessed the prognostic significance of *FoxP3* status on survival in AML. While survival was significantly reduced in *TRDV2*^LO^ leukemias that were also *FoxP3*^HI^ rather than *FoxP3*^LO^, no such effect was observed when comparing *TRDV2*^HI^ *FoxP3*^HI^ or *TRDV2*^HI^ *FoxP3*^LO^ leukemias (Figure S7G). This suggests that FoxP3 is not detrimental to disease outcome when Vδ2 γδ T cells are present. While these data do not definitively establish that γδ[T2] cells are present in human cancer, they point strongly toward this possibility in TGF-β-rich cancers such as AML, and link this with improved disease outcomes.

## Discussion

We have shown here that TGF-β1 operates through multiple mechanisms to enhance the antitumor activity of *ex vivo* expanded Vγ9Vδ2 T cells. Culture in the presence of TGF-β led to a clear increase in viability, associated with a favorable ratio of anti-apoptotic to pro-apoptotic protein expression.^37^ Despite the known inhibitory effect of TGF-β1 on Vγ9Vδ2 T cell proliferation,^16^^,^^37^ the cell yield from γδ[T2] cultures was increased. γδ[T2] cells were also less differentiated, a finding linked to superior clinical outcomes using engineered αβ T cells.^38^ Moreover, γδ[T2] cells were uniquely desensitized to two key immunosuppressive factors in the tumor microenvironment, namely TGF-β itself and PGE_2_. PGE_2_ potently suppresses the proliferation, cytokine production, and cytotoxic capacity of γδ T cells.^39^

Given its pleiotropic actions, several additional factors are likely to contribute to the superior antitumor activity of TGF-β-conditioned Vγ9Vδ2 T cells, and many candidates were identified by our RNA-seq analysis. These include the upregulation of Ephrin A1 and A4 receptors, which favor memory T cell migration across high endothelial venules.^40^ CD300A inhibits cellular responses upon binding to lipids exposed during cell death^41^ and was downregulated in γδ[T2] cells, potentially protecting them from the inhibitory effects of apoptotic or necrotic cells. *LPAR6* encodes a receptor that binds the immunosuppressive intermediate, adenosine, and was also reduced in γδ[T2] cells. Finally, TGF-β has been shown to repress mammalian target of rapamycin (mTOR) signaling to promote a less exhausted T cell metabolic state.^42^ We observed that γδ[T2] cells expressed gene sets that favored enhanced glucose generation, consumption, and fatty acid metabolism. Consequently, further metabolic characterization of these cells is warranted.

Although short-term killing activity was unchanged, γδ[T2] cells consistently achieved superior cytolytic function at 24 h. In the case of E-cadherin^POS^ solid tumors, this resulted in part from CD103 upregulation, as reported previously.^15^ By contrast, blocking studies implicated CD11a in the increased killing of E-cadherin^NEG^ leukemic cells, consistent with the known role of LFA-1 in Vγ9Vδ2 T cell cytotoxicity,^43^ and the expression of both LFA-1^44^ and its main ligand (ICAM-1) on AML^45^ and other tumors. Our RNA-seq data confirmed that a number of effectors of cytotoxic activity were transcriptionally downregulated in γδ[T2] cells, including perforin and granzymes A, K, and M, while there was no change in other death effectors (e.g., FasL, TRAIL, granzyme B). These findings argue that γδ[T2] cells achieve enhanced killing in part through more efficient integrin-mediated target cell interaction, rather than potentiation of their pro-apoptotic machinery. The potential contributory roles of insensitivity to TGF-β and PGE_2_ and cytokine-mediated cell death (e.g., necroptosis induced by TNF-α/IFN-γ, both of which are overproduced by activated γδ[T2] cells) also warrant further study. It should also be noted that all of the data shown in this study were generated using immortalized cell lines, providing proof of concept. Further testing against patient-derived leukemic and tumor xenografts would be useful, although heterogeneity of these models requires consideration.

While γδ[T2] cells were preferentially cytotoxic against transformed cells, some background killing of immortalized non-transformed cells was observed, incurring a risk of autoimmunity. Thus far, clinical experience with Vγ9Vδ2 T cell immunotherapy has proven safe, although the enhanced cytolytic activity of γδ[T2] cells may also increase the potential for toxicity. Given the frequent overproduction of chemokines by solid tumors, safer use of γδ[T2] cells may be achieved through the expression of an appropriate chemokine receptor to preferentially direct the cells into malignant rather than healthy tissue. Using this strategy, we found that both the efficacy and safety of chimeric antigen receptor (CAR) T cell immunotherapy were enhanced.^46^

Given their enhanced bone marrow trafficking capacity, we posited that adoptive immunotherapy using γδ[T2] cells would be effective for hematologic malignancies. We observed the strong therapeutic activity of these cells in leukemic model systems. However, the altered biodistribution of γδ[T2] cells may have compromised their use in solid tumor immunotherapy, despite the frequent production of CXCR4 ligands^47^ and expression of E-selectin on the vasculature of both human and mouse carcinomas.^48^ This limitation could be circumvented by regional (i.p.) delivery in models of epithelial ovarian cancer (EOC), enabling the superior anticancer activity of γδ[T2] cells to again be harnessed.

Under some circumstances, TGF-β1 confers a Treg phenotype on γδ T cells.^13^^,^^37^^,^^49^, ^50^, ^51^ We observed such a gene signature in γδ[T2] cells using GSEA. More important, however, γδ[T2] cells lacked functional Treg activity. Where TGF-β1 has promoted γδ T-reg differentiation, cultures included fetal calf serum, IL-15, and/or vitamin C, none of which were used here. Recently, it was shown that TGF-β-conditioned Vγ9Vδ2 T cells exhibited transient and low-level FoxP3 expression without demethylation of the FoxP3 Treg-specific demethylated region or convincing Treg function, unless vitamin C was also present.^51^

In comparing Vγ9Vδ2 tumor-infiltrating lymphocytes (TILs) across cancers, we and others^52^ found that their abundance was greatest in AML. Our bioinformatic analyses suggest that γδ TILs found in AML resemble γδ[T2] cells and are associated with improved survival. While thymomas also contain a large number of Vγ9Vδ2 TILs, they do not share a γδ[T2]-like phenotype or have a similar impact on outcomes. This may be the result of the lower abundance of *TGFB1* transcripts in this tumor. Although clear evidence of an IL-9 autocrine loop was found in γδ[T2] cells, this was not implicated in their enhanced superior *in vitro* function, either against leukemic or solid tumor cell lines. Nonetheless, analysis of TCGA datasets in AML provide clinical evidence that an IL-9-rich environment in combination with Vγ9Vδ2 TILs was linked with the best prognosis, suggesting that this cytokine contributes importantly *in vivo* to γδ[T2] cell biology. On a cautionary note, IL-9 itself has been linked to pro-leukemic effects in some settings.^53^

While our data provide support for the use of γδ[T2] cells in the immunotherapy of a range of cancers, AML represents a particularly attractive option for clinical evaluation of this strategy. A substantial proportion of AML samples are naturally susceptible to Vγ9Vδ2 T cells.^54^^,^^55^ Consequently, AML patients in disease remission may benefit from consolidation immunotherapy using healthy donor-derived allogeneic γδ[T2] cells. Given the variability in the yield, immunophenotype, function, and diversity of these cells,^56^, ^57^, ^58^ carefully selected healthy donors will be required for the production of cell products. Conditioning of patients with fludarabine, Ara-C, and ZOL would benefit from the combined lymphodepleting, antileukemic, and γδ T cell-sensitizing actions of these drugs, providing an intriguing platform for the evaluation of this immunotherapeutic strategy. To aid in clinical development, there is a need to define predictive biomarkers that correlate with the enhanced antileukemic potency of these heterogeneous cell products, a task that will require considerable further study.

### Limitations of the study

We have not dissected the effects of TGF-β on non-δ2 γδ T cells present when these cells are expanded using antibody activation. Moreover, the compatibility of this platform with genetic targeting approaches such as chimeric antigen receptors remains untested.

## STAR★Methods

### Key resources table

**Table:** 

REAGENT or RESOURCE	SOURCE	IDENTIFIER
**Antibodies (mouse anti-human unless otherwise indicated)**		

IgG1 – FITC	Beckman Coulter	Cat# IM0639U, RRID:AB_130990
IgG1 – APC	R&D Systems	Cat# IC002A, RRID:AB_357239
IgG1 - PE	BioLegend UK	Cat# 400112, No RRID available
IgG1 – PE/Cy7	BioLegend UK	Cat# 400126, No RRID available
IgG1 – BV605	BioLegend UK	Cat# 400162, No RRID available
IgG1 - PerCP	BioLegend UK	Cat# 400148, No RRID available
IgG1 – AF647	BioLegend UK	Cat# 400130, No RRID available
IgG2B - APC	R&D Systems	Cat# IC0041A, RRID:AB_357246
IgG2A - PE	BioLegend UK	Cat# 400202, No RRID available
Rat IgM-PE	BioLegend UK	Cat# 400808, No RRID available
TCR Pan γδ purified (11F2)	BD Biosciences	Cat# 347900, RRID:AB_400356
TCR Pan γδ - FITC (B1)	BD Biosciences	Cat# 559878, RRID:AB_397353
TCR Pan γδ – PE (B1.1)	eBioscience	Cat# 12-9959-42, RRID:AB_1603300
TCR Vγ9 – APC (B3)	BioLegend UK	Cat# 331310, RRID:AB_2057504
TCR Vγ9 (7A5)	Life Technologies	Cat# TCR1720, RRID:AB_417089
TCR Vδ2 – APC (B3)	BioLegend UK	Cat# 331417, RRID:AB_2687323
CD2 – APC (RPA-2.10)	BioLegend UK	Cat# 300213, RRID:AB_10900259
CD3 – APC (OKT3)	BioLegend UK	Cat# 317317, RRID:AB_1937213
CD3 – PE/ Cy7 (OKT3)	BioLegend UK	Cat# 317333, RRID:AB_2561451
CD3 – APC/Cy7 (HIT3a)	BioLegend UK	Cat# 300318, RRID:AB_314054
CD3 – APC/Cy7 (SK7)	BioLegend UK	Cat# 344817, RRID:AB_10644011
CD4 – FITC/ AF700 (RPA-T4)	BioLegend UK	Cat# 300506, RRID:AB_314074
CD4 – FITC (A161A1)	BioLegend UK	Cat# 357405, RRID:AB_2562356
CD4 – APC (OKT4)	BioLegend UK	Cat# 317416, RRID:AB_571945
CD8 – PE/Cy7 (SK1)	BioLegend UK	Cat# 344712, RRID:AB_2044008
CD8a – PE (RPA-T8)	BioLegend UK	Cat# 301064, RRID:AB_2564167
CD11a – APC (HI111)	BioLegend UK	Cat# 301212, RRID:AB_314150
CD16 – APC (3G8)	BioLegend UK	Cat# 302012, RRID:AB_314212
CD25 – PE (M-A251)	BioLegend UK	Cat# 356103, RRID:AB_2561860
CD27 – PE (M-T271)	BioLegend UK	Cat# 356405, RRID:AB_2561824
CD27 – PE (LG.3A10)	BioLegend UK	Cat# 124209, RRID:AB_1236464
CD28 – PE/ (CD28.2)	BioLegend UK	Cat# 302908, RRID:AB_314310
CD45 – FITC (HI30)	BioLegend UK	Cat# 304054, RRID:AB_2564154
CD45RO – APC (UCHL1)	BioLegend UK	Cat# 983102, RRID:AB_2650651
CD45RO – PE/Cy7 (UCHL1)	BioLegend UK	Cat# 304230, RRID:AB_11203900
CD45RA – BV605/ APC (HI100)	BioLegend UK	Cat# 304150, RRID:AB_2564158
CD57 – APC (HCD57)	BioLegend UK	Cat# 322314, RRID:AB_2063199
CD62L – PerCP/Cy5.5 (DREG-56)	BioLegend UK	Cat# 304824, RRID:AB_2239105
CD69 – APC (FN50)	BioLegend UK	Cat# 310909, RRID:AB_314844
CD70 – PE	BD Biosciences	Cat# 555835, RRID:AB_396158
CD103 (ITGAE) -	Biolegend UK	Cat# 350216 RRID:AB_2563907
CD112 (Nectin-2) – APC (TX31)	BioLegend UK	Cat# 337412, RRID:AB_2565730
CD127 – PE	BioLegend UK	Cat# 351304, RRID:AB_314817
CD129 – PE (AH9R7))	BioLegend UK	Cat# 310404, RRID:AB_314817
CD155 (PVR) – APC (SKII.4)	BioLegend UK	Cat# 337618, RRID:AB_2565815
CD178 (Fas-L) – PE (NOK-1)	BioLegend UK	Cat# 306407, RRID:AB_2100664
CD184 (CXCR4) – APC (12G5)	BioLegend UK	Cat# 306510, RRID:AB_314616
CD197 (CCR7) – BV605 (G043H7)	BioLegend UK	Cat# 353223, RRID:AB_11124325
CD197 CCR7-FITC (FAB197F-100)	R&D Systems	Cat# FAB197F, RRID:AB_2259847
CD223 (LAG-3) – AF 647 (11C3C65)	BioLegend UK	Cat# 369303, RRID:AB_2566479
CD226 (DNAM-1) – APC (DX11)	Miltenyi Biotec	Cat# 130-092-477, RRID:AB_615073
CD244 – APC (2B4)	BioLegend UK	Cat# 329512, RRID:AB_2072861
CD277 (BT3.1) – PE (BT3.1)	BioLegend UK	Cat# 342704, RRID:AB_2290526
CD300A – PE (MEM-260)	ThermoFisher	Cat A15778, RRID:AB_2534558
CD314 (NKG2D) – PE (1D11)	BioLegend UK	Cat# 320805, RRID:AB_492961
CD314 (NKG2D) – PE/Cy7 (1D11)	BioLegend UK	Cat# 320811, RRID:AB_2133275
CD335 (NKp46) – APC (9E2)	BioLegend UK	Cat# 331917, RRID:AB_2561649
CD336 (NKp44) – APC (P44-8)	BioLegend UK	Cat# 325109, RRID:AB_2149433
CD337 (NKp30) – APC (P30-15)	BioLegend UK	Cat# 325209, RRID:AB_2149450
CD366 (Tim-3) – APC (F38-2E2)	BioLegend UK	Cat# 345011, RRID:AB_2561717
KLRG1 -PE	BioLegend UK	Cat# 368609, RRID:AB_2572136
Anti-EGF antibody	BioLegend UK	Cat# 679502, RRID:AB_2566190
9e10 anti-myc antibody	Prepared in house	Hybridoma supernatant. RRID:AB_558470
PTGER2-PE	Abcam	Cat# ab92755. RRID:AB_10563848
Beta-actin-HRP	BioLegend UK	Cat# 643808, RRID:AB_2734515
Rat anti-human/mouse CLA-PE	BioLegend UK	Cat# 321312, RRID:AB_2565589
FoxP3 - APC	Miltenyi Biotec	Cat# 130-125-580. No RRID available
Simultest CD3-FITC / CD16+56-PE	BD Biosciences	Cat# 342403, RRID:AB_2868771
Cleaved Caspase 3-AF488	R&D systems	Cat# IC835G, RRID:AB_2243951
Normal goat IgG	R&D systems	Cat# AB-108-C, RRID:AB_2868771
Mouse IgG1 LEAF	BioLegend UK	Cat# 400166, No RRID available
Mouse IgG2a LEAF	BioLegend UK	Cat# 401508, No RRID available
Goat IL-9 blocking antibody	Novus Biologicals	Cat# AF209, RRID:AB_2296123
CD11a blocking antibody	BioLegend UK	Cat# 301233, RRID:AB_2832576
CD103 blocking antibody	BD Biosciences / BioLegend UK	Cat# IM0318, RRID:AB_558012 Cat# 250202, RRID:AB_10639864

**Bacterial and virus strains**		

Competent *E. coli*	Sigma	Cat# CMC0001
SFG retroviral vector	Dr Michel Sadelain, MSKCC	N/A

**Biological samples**		

Human anticoagulated blood	Healthy donors	N/A
Primary human pulmonary endothelial cells	Promocell	Cat# C-12281

**Chemicals, peptides, and recombinant proteins**		

7-AAD	Sigma	Cat# SML1633
Annexin-V – PE	ThermoFisher	Cat# 88-8102-72.
Annexin-V – APC	ThermoFisher	Cat# 88-8007-72.
Ara C	Hospira	N/A
Camptothecin	Sigma	Cat# C9911
ECL	ThermoFisher	Cat# 32209
EHS matrix extract	Sigma Aldrich	Cat# 126-2.5,
Ficoll-Paque Plus	GE Heathcare	Cat# 17-1440-03
FBS	Sigma Aldrich	Cat# F0804
Glutaraldehyde	Sigma Aldrich	Cat# 340855
Human AB serum	Sigma Aldrich	Cat# H4522
IL-2 (Proleukin)	Novartis	N/A
IL-9	Peprotech	Cat# 200-09
Luciferin	Regis Technologies	Cat# 115144-35-9
Methylene Blue	Sigma Aldrich	Cat# M4159
MTT	Sigma	Cat# M5655
Pamidronic acid	Wockhardt	N/A
PGE_2_	Sigma	Cat# P6532
Propidium iodide	BD PharMingen	Cat# 556463
RetroNectin	Takara	Cat# T202
Rh E-selectin (CD62)-Fc chimera	R&D Systems	Cat# 724-ES-100
RIPA buffer	Abcam	Cat# ab156034
TGF-β1	BioTechne	Cat# 240-B,
TRIzol	ThermoFisher	Cat# 15596026
Zometa	Novartis	N/A

**Critical commercial assays**		

Human apoptosis array kit	R&D Systems	Cat# ARY009,
Human luminex 30-plex cytokine array kit	ThermoFisher	Cat# LHC6003
PlasmoTest™ mycoplasma test	Invivogen	Cat# rep-pt1,
Zombie NIR (TM) Viability kit	BioLegend	Cat# 423106,
BCA protein quantification kit	ThermoFisher (Pierce)	Cat# 23225
Ribozero	Illumina	Cat# 20040526
NEBNext	NEB	Cat# E6040
Human IFNγ ELISA	eBiosciences	Cat# 88-7316
Human IL-9 ELISA	eBiosciences	Cat# 88-7958
Human TNFα ELISA	biolegend	Cat# 430201
Human IL-10 ELISA	Bio-Techne	Cat# DY217B
Human IL-2 ELISA	eBiosciences	Cat# 88-7025

**Deposited data**		

RNA-seq data	NCBI Gene expression omnibus (GEO)	GEO: GSE171973

**Experimental models: cell lines**		

Jurkat E6.1	Dr Linda Barber, King’s College London	ATCC Cat# TIB-152 RRID CVCL_0367
KG1	Dr Linda Barber, King’s College London	ATCC Cat# CCL-246 RRID CVCL_0374
U937	Dr Linda Barber, King’s College London	ATCC Cat# CRL-1593.2 RRID CVCL_0007
MDA-MB-231	Breast Cancer Now Research Unit, King’s College London	ATCC Cat# HTB-26 RRID CVCL_0062
MDA-MB-468	Breast Cancer Now Research Unit, King’s College London	ATCC Cat# HTB-132 RRID CVCL_0419
BT-20	Breast Cancer Now Research Unit, King’s College London	ATCC Cat# HTB-19 RRID CVCL_0178
SKOV-3	PerkinElmer	Cat# BW119276 RRID CVCL_0532
Kuramochi	Japanese Collection of Research Bio-resources Cell Bank	Cat# JCRB0098
Ovsaho	Japanese Collection of Research Bio-resources Cell Bank	Cat# JCRB1046 RRID CVCL_3114
MRC-5	American type Culture Collection	ATCC Cat# CRL-171 RRID CVCL_0440
H29	Dr Michel Sadelain, Memorial Sloan Kettering Cancer Centre, New York, USA.	N/A
PG13	European Collection of Authenticated Cell Culture	Cat# 95110215 RRID CVCL_4273
SH-SY5Y	Dr Ximena Montano, King’s College London	ATCC Cat# CRL-2266 RRID CVCL_0019
9e10 hybridoma	ECACC	Cat# 85102202

**Experimental models: organisms/strains**		

NSG	Charles River	N/A
SCID-Beige	Charles River	N/A

**Recombinant DNA**		

SFG ITGAE	Genscript	N/A
SFG GFP	Dr Michel Sadelain, MSKCC	N/A
SFG RFP ffLuc	Genscript	N/A

**Software and algorithms**		

Prism 9.0	GraphPad	https://www.graphpad.com/scientific-software/prism/
ImageJ	Schneider et al., 2012^72^	https://imagej.nih.gov/ij/
FlowJo v9	FlowJo, LCC, BD Biosciences	https://www.flowjo.com/solutions/flowjo/downloads/v9
Cellquest Pro v5	BD Biosciences	N/A
VivoQuant	Invicro	http://www.vivoquant.com
fastQC	Andrews et al., 2010^73^	https://www.bioinformatics.babraham.ac.uk/projects/fastqc/,
trimmomatic	Bolger et al., 2014^61^	http://www.usadellab.org/cms/?page=trimmomatic
Hisat2	Kim et al., 2015^62^	http://daehwankimlab.github.io/hisat2/
Ensembl	Yates et al., 2020^63^	http://www.ensembl.org//useast.ensembl.org/index.html?redirectsrc=//www.ensembl.org%2Findex.html
Htseq-count	Anders et al., 2015^64^	https://htseq.readthedocs.io/en/release_0.11.1/count.html
DESeq2	Love et al., 2014^65^	https://bioconductor.org/packages/release/bioc/html/DESeq2.html
fdrtool	Strimmer, 2008^67^	https://cran.r-project.org/web/packages/fdrtool/index.html
Xena Browser	Goldman et al., 2020^68^	https://xenabrowser.net
Kmplot	Nagy et al., 2021^74^	https://kmplot.com/analysis/
GSEA	Subramanian et al., 2005^69^; Mootha et al., 2003^75^	https://www.gsea-msigdb.org/gsea/index.jsp
PRECOG	Gentles et al., 2015^2^	https://precog.stanford.edu
Living Image 4.7.3	PerkinElmer	https://www.perkinelmer.com/lab-products-and-services/resources/in-vivo-imaging-software-downloads.html

**Other**		

Antibiotic Antimycotic	ThermoFisher	Cat# 15240096
DMEM	Lonza	Cat# BE12-709F
Endothelial cell Growth Medium Kit	Promocell	Cat# C-22120
Glutamax	ThermoFisher	Cat# 35050061
RPMI 1640 with L-Glutamine	Lonza	Cat# BE12-702F
TexMACS GMP media	Miltenyi Biotec	Cat# 170-076-307

### Resource availability

#### Lead contact

Further information and requests for resources and reagents should be directed to and will be fulfilled by the lead contact, John Maher (john.maher@kcl.ac.uk).

#### Materials availability

Reagents generated in this study will be made available on request, but we may require a payment and/or a completed Materials Transfer Agreement if there is potential for commercial application.

### Experimental model and subject details

#### Mice

Experiments were performed with mice aged 6-10 weeks. All *in vivo* experimentation adhered to UK Home Office guidelines, as specified in project license numbers 70/7794 and P23115EBF and was approved by the King’s College London animal welfare and ethical review body (AWERB). SCID Beige (CB17.Cg-Prkdc^scid^Lyst^bg-J^/Crl) and Nod SCID γc^null^ (NSG; NOD.Cg-Prkdc^scid^ Il2rg^tm1Wjl^/SzJ) mice were purchased from Charles River Laboratories. Both male and female mice were used in equal numbers except in models of breast and ovarian cancer in which female mice were used. An equivalent number of male and female donors were used otherwise and we did not detect any clear impact of gender on the findings of our study. Animals were housed in individually ventilated cages within the Biological Services Units at King’s College London. Mice were randomly allocated to experimental groups based on similar average tumor burden prior to treatment.

#### Cell lines and tissue culture

Tumor, leukemic and immortalized cell lines were obtained from US or European Cell Banks or stocks that were validated using short tandem repeat analysis and were subject to regular mycoplasma testing. Cell lines were maintained in either Dulbecco’s Modified Eagle’s Medium or RPMI 1640 supplemented with 10% FCS, l-glutamine and antibiotic-antimycotic solution. Primary human pulmonary endothelial cells were maintained in endothelial cell growth medium. Cells were engineered to co-express ffLuc/RFP or a GFP reporter gene using an SFG retroviral vector as described.^59^ Cells were maintained at 37°C in a humidified atmosphere of 5% CO_2_.

#### Human study oversight

Blood samples were also obtained from healthy donors following approval of the study protocol by a National Health Service Research Ethics Committee (09/H0804/92 and 18/WS/0047).

### Method details

#### Culture and genetic modification of primary human Vγ9Vδ2 T cells

After isolation by density gradient separation, PBMC were plated at a density of 3x10^6^ cells/ml in TexMACS SFM supplemented with GlutaMax and antibiotic-antimycotic solution. Where specifically indicated, medium also contained 10% human AB serum. Activation of Vγ9Vδ2 T cells was achieved using ZOL (1 μg/ml) or immobilized pan-γδ TCR antibody (0.8μg/mL). On the day of activation, IL-2 (100U/ml) and, in some cases, TGF-β (5ng/mL) and/ or IL-9 (concentrations specified) were added. Where indicated, cultures were supplemented with a blocking IL-9 antibody (neutralizing concentration 2-5μg/mL per 5ng/mL IL-9; https://www.novusbio.com/products/il-9-antibody_af209#datasheet, accessed 12-09-2017). Cytokines, inhibitors and/or blocking antibodies were replenished every 2-3 days with addition of medium as appropriate over a total culture period of at least 15 days. Retroviral transduction of Vγ9Vδ2 T cells was performed 7 days after activation using PG13-derived viral particles, pre-loaded on RetroNectin coated plates.^60^ The *ITGAE* gene was synthesized and was fused via a downstream furin cleavage site (RRKR) and *Thosea Asigna* (T2A) ribosomal skip peptide to a membrane anchored epitope tag in which a human CD8α leader peptide was joined to a 9e10 myc epitope (EQKLISEEDL) followed by codons 114-180 of human CD28 to achieve membrane anchoring. This construct was delivered to γδ T cells using the SFG retroviral vector.^60^

#### Protein analysis

Flow cytometric analysis was performed using FACScalibur cytometer with on an LSR FORTESSA analyzer, recording at least 5 × 10^5^ events. Compensation settings were established using single stained samples. Intracellular staining was performed after fixation and permeabilization of cells with 4% paraformaldehyde and 0.1% saponin. Where viability of cells was assessed following camptothecin exposure, 10^6^ cells were pre-exposed to camptothecin (12μM) for 6h at 37°C prior to analysis. Cells were first gated based on forward (FSC-A) and side (SSC-A) scatter (measuring cell size and granularity respectively) to exclude debris. Single cells were then selected using SSC-A versus SSC-W parameters. Dead cells were excluded using a viability stain.

To test E-selectin binding, cells were incubated with 1μg recombinant E-selectin-Fc in the presence of FACS buffer (0.5% BSA in PBS) plus 5mM EDTA or 5mM Ca^2+^ (as indicated) overnight at 4°C. On the next day, anti-human IgG Fc-FITC secondary antibody was added for 30 m at 4°C prior to washing and analysis by flow cytometry.

Human cytokines were quantified by ELISA or Luminex 30-plex cytokine array kit, as described by the manufacturers, using CLARIOSTAR or Flexmap 3D platforms respectively. Expression of proteins involved in apoptotic cell death was determined using a Proteome Profiler Human apoptosis array kit, as recommended by the manufacturers and analyzed using ImageJ. Cell lysates from matched day 15 cells were prepared for immunoblotting analysis using a RIPA buffer as per manufacturer’s instructions. 10μg of cell lysate, quantitated using the BCA assay, was reduced and separated by SDS-PAGE (100V) before being transferred onto PVDF membrane. Membranes were blocked in 5% milk powder in tris-buffered saline with 0.1% tween 20 (TBST) for 1h at 4°C, before being probed with primary antibodies (at concentrations recommended by the manufacturer) overnight at 4°C. Membranes were washed in TBST before being probed with appropriate secondary horseradish peroxidase-labeled antibodies for 1h at RT. Membranes were washed with TBST and developed using ECL. Pixel density in relevant bands was quantified in captured electronic Images using ImageJ software.

To test effect of PGE_2_ on IFN-γ production, γδ[2] or γδ[T2] cells were co-cultivated with ffLuc-expressing U937 and KG-1 for 72h at 1:1 E:T ratio in the presence of the indicated concentration of PGE_2_. IFN-γ was measured in harvested supernatant by ELISA. Percentage inhibition of IFN-γ production was calculated using the formula:

[IFN-γ] in the absence of PGE_2_ - [IFN-γ] in the presence of PGE_2_ x 100% divided by [IFN-γ] in the absence of PGE_2_

#### Cytotoxicity assays

Co-cultivation assays between T cells and 1 × 10^4^ target cells were established for intervals and at effector to target (E:T) ratios as specified in individual experiments. Where indicated, target cell destruction was quantified by *in vitro* MTT or luciferase assays, as described.^59^ Alternatively, viability of target cells in monolayer cultures was monitored by real time impedance measurement using an xCELLigence RTCA MP (ACEA Biosciences, San Diego CA), as recommended by the manufacturers. Where indicated, target cells were sensitized with indicated concentrations of ZOL, PAM and/ or Ara C for 24h prior to addition of Vγ9Vδ2 T cells. Residual viable cells were normalized to tumor or untreated control cells that were cultured alone (set at 100%).

In the case of cytotoxicity assays using SH-SY5Y cells, tumor cells were sensitized with 1 μg/mL ZOL for 24h, washed once in serum-free media. γδ[2] or γδ[T2] cells were added at a 5:1 E:T ratio. After 24h, media was aspirated from wells and cells were fixed with 4% glutaraldehyde for 20 minutes at room temperature. Cells were washed twice with PBS and subsequently stained with 0.05% methylene blue for 20 minutes at room temperature, with gentle shaking. Cells were washed three times in a tray of running water then air-dried upside down, overnight at room temperature. The following day, methylene blue dye was extracted by addition of 3% HCl and incubation for 30 minutes with gentle shaking, at room temperature. Cell viability was determined by measuring absorbance at 655nm on the FLUOstar Omega plate reader (BMG Labtech). All OD values were corrected by subtraction of background values generated using the media alone control, and viable cell numbers were calculated using a standard curve prepared with tumor cells alone.

To test the effect of CD11a blockade on cytotoxicity, KG-1 cells were plated at 1x10^5^ cells in 24 plates in TexMACS media. ZOL (1mg/mL) was applied to appropriate wells for 24h. γδ[2] or γδ[T2] cells were added at a 5:1 E:T ratio. After 24h, all cells were removed and stained with a fixable viability dye and anti-CD3. Live KG-1 cells were defined by lack of uptake of the viability dye and negative staining for anti-CD3. Percentages of live KG-1 cells remaining after co-culture with a CD11a neutralizing antibody were corrected against live KG-1 cell percentages when using an isotype control.

To test effect of PGE_2_ on cytotoxicity, γδ[2] or γδ[T2] cells were co-cultivated with ffLuc-expressing U937 and KG-1 for 72h at 1:1 E:T ratio in the presence of the indicated concentration of PGE_2_. Leukemic cell viability was assessed by luciferase assay, normalized to leukemic cells alone (100% viability). Percentage inhibition of cytotoxicity or was calculated using the formula:

% cytotoxicity in the absence of PGE_2_ - % cytotoxicity in the presence of PGE_2_ x 100 divided by % cytotoxicity in the absence of PGE_2_

#### *In vivo* measurement of anti-leukemic and anti-tumor activity

Tumor and leukemic cells were transduced with SFG ffLuc/RFP^59^ and were purified by flow sorting prior to engraftment in mice as indicated for individual experiments. Mice with similar average tumor burden were randomly assigned to groups for blinded treatment with the indicated agents. Bioluminescence imaging was performed as described.^59^ In all experiments, animals were inspected daily and weighed weekly. Mice were culled if symptomatic as a result of tumor progression or weight loss of ≥ 20%.

#### Imaging ^89^Zr labeled Vγ9Vδ2 T cells using PET-CT

Radio-labeling of *ex vivo* expanded Vγ9Vδ2 T cells with [^89^Zr]Zr(oxinate)_4_ was performed as described.^29^ In brief, 26 × 10^6^ T cells were washed twice with PBS and re-suspended in 4 mL PBS to which 100μl of [^89^Zr]Zr(oxinate)_4_ (70-80MBq dissolved in 30% dimethylsulfoxide) was added. Following incubation for 25 minutes at room temperature with regular gentle mixing by swirling, cells were washed with PBS and re-suspended in 300 μL PBS at a final cell density of 8.9 × 10^7^ cells per mL.

Animals were injected i.v. with 5 × 10^6^ 89Zr-labeled Vγ9Vδ2 T cells (5MBq). Mice were imaged by PET-CT for 60 minutes at 24 and 48 hours after T cell injection. Images were reconstructed and activity within regions of interest (ROI) were calculated. Bio-distribution analysis was performed by *ex vivo* tissue gamma counting.

#### RNA sequencing analysis

ZOL-activated γδ[2] and γδ[T2] cells were expanded from 3 separate healthy donors as described above. On days 9 and 15 of the culture, Vγ9Vδ2 T cells were flow-sorted to purity and RNA extracted using TRIzol. Ribosomal RNA was removed using Ribo-Zero and libraries were prepared using NEBNext. RNA-Seq was performed with a minimum sequencing depth of 100,000 reads. The quality of the tags was inspected with fastQC (https://www.bioinformatics.babraham.ac.uk/projects/fastqc/, accessed 15/06/2020). Raw reads were trimmed and filtered to remove adaptor contamination and poor-quality bases using trimmomatic.^61^ The resulting FASTQ were mapped to the GRCh38 assembly of the human genome using Hisat2 with default parameters.^62^ The number of reads mapping to the genomic features annotated in Ensembl^63^ with a MAPQ score higher than or equal to 30 was calculated for all samples using htseq-count with default parameters.^64^

Features with no mapped reads in at least one sample or with less than 10 reads on average across all samples were not included in downstream analyses. Differential gene expression analysis between sample groups were performed in R using the Wald test as implemented in the DESeq2 package.^65^ *p* values were adjusted for multiple testing according to the Benjamini and Hochberg procedure.^66^ The raw and adjusted *p* values were re-estimated empirically with fdrtool,^67^ when the histograms of the initial *p* value distributions showed that the assumptions of the Wald test were not met.

#### The Cancer Genome Atlas Genomic Data Commons (TCGA GDC) determination of the γδ[T2] cell signature and survival analysis

TCGA GDC datasets were downloaded from Xena Browser (https://xenabrowser.net, accessed May 20^th^, 2020) and TCR transcript survival and correlation analysis performed on the Xena Functional Genomics Explorer.^68^ We selected the 24 differentially expressed genes with the lowest estimated false discovery rate (FDR = 8.44e-14) from the RNA-Seq analysis of γδ[2] and γδ[T2] samples, thereby generating a γδ[T2] cell signature (Figure 6B). Using RNA-Seq TCGA datasets derived from a range of cancer types, the impact of γδ[T2] cell signature on survival was tested and downloaded using Kmplot (Semmelweis University, Budapest; https://kmplot.com/analysis/, accessed May 22^nd^, 2020).

#### Gene set enrichment analysis (GSEA)

GSEA (v4.1.0) was downloaded from the Broad Institute.^69^ Expression data was inputted as mean (of the 3 donors) transcripts per million (TPM) for class A (γδ[2] cells) and class B (γδ[T2] cells). The analysis was run on the following MSigDb gene sets databases,^70^ with 1000 permutations and no collapsing: h.all.v7.2.symbols.gmt, c1.all.v7.2.symbols.gmt, c2.all.v7.2. symbols.gmt, c2.cpg.v7.2.symbols.gmt, c2.cp.v7.2.symbols.gmt, c2.cp.biocarta. v7.2.symbols.gmt, c2.cp.kegg.v7.2.symbols.gmt, c2.cp.pid.v7.2.symbols.gmt, c2.cp.reactome.v7.2.symbols.gmt, c2.cp.wikipathways.v7.2.symbols.gmt, c3.all. v7.2.symbols.gmt, c4.all.v7.2.symbols.gmt, c5.all.v7.2.symbols.gmt, c6.all. v7.2.symbols.gmt, c7.all.v7.2.symbols.gmt, c8.all.v7.2.symbols.gmt. The most significant results are included in Figure 6 and Table S3.

#### PREdiction of Clinical Outcomes from Genomic profiles (PRECOG)

The γδ[T2] cell signature was used to calculate survival outcomes using prediction of clinical outcomes from genomic profiles (PRECOG),^2^ a tool which calculates prognostic survival scores through a meta-analysis by integrating 165 cancer gene expression datasets and clinical outcomes across ∼26,000 patients presenting with 39 malignancies. PRECOG scores for genes within the γδ[T2] cell signature with a negative z score (i.e., downregulated in γδ[T2] cells compared to γδ[2] cells) were transformed by multiplying by −1. Other Vγ9Vδ2 T cell marker genes available on PRECOG were subsequently included to evaluate survival outcome predictions based on the presence of these cells. Downstream analyses and plots were conducted in R (version 3.6.1), using the tidyverse^71^ packages (version 1.3.0) as well as the plotly package (version 4.9.2.1).

### Quantification and statistical analysis

Normality of all experimental data was tested using the Shapiro-Wilk test prior to statistical analysis. Statistical analysis was performed using two-tailed Student’s t test, one-way or two-way ANOVA (normally distributed datasets), or Wilcoxon signed rank test (other datasets). To test correlation between IL-9 production by γδ[2] and γδ[T2] cells from individual donors a Spearman test was performed (data not normally distributed). Survival data were analyzed using the Log-rank (Mantel-Cox) test. All statistical analysis was performed using GraphPad Prism version 9.0 (GraphPad software, San Diego, CA) or Excel for Mac 2020 (Berkshire, UK).

## Data Availability

RNA-seq data were deposited at NIH Gene Expression Omnibus (GEO) and are publicly available as of the date of publication. There was no new code developed as part of this study. Any additional information required to re-analyze the data reported in this work paper is available from the lead contact upon request.

## Supplemental information

Document S1. Figures S1–S7

Table S1. Differentially expressed genes between γδ[2] and γδ[T2] cells, day 9, related to Figure 6

Table S2. Differentially expressed genes between γδ[2] and γδ[T2] cells, day 15, related to Figure 6

Table S3. Gene set enrichment analysis, related to Figure 6

Document S2. Article plus supplemental information
