# Supplementary material for: TGF-β1 potentiates Vγ9Vδ2 T cell adoptive immunotherapy of cancer
Source: Cell Rep Med. 2021 Dec 21;2(12):100473. doi: 10.1016/j.xcrm.2021.100473 (PMC8714942; doi:10.1016/j.xcrm.2021.100473)
Supplement: Document S1. Figures S1–S7 [file mmc1.pdf]

Cell Reports Medicine, Volume *2*

Supplemental information

TGF-β1 potentiates Vγ9Vδ2 T cell adoptive immunotherapy of cancer

Richard E. Beatson, Ana C. Parente-Pereira, Leena Halim, Domenico Cozzetto, Caroline Hull, Lynsey M. Whilding, Olivier Martinez, Chelsea A. Taylor, Jana Obajdin, Kim Ngan Luu Hoang, Benjamin Draper, Ayesha Iqbal, Tom Hardiman, Tomasz Zabinski, Francis Man, Rafael T.M. de Rosales, Jinger Xie, Fred Aswad, Daniela Achkova, Chung-Yang Ricardo Joseph, Sara Ciprut, Antonella Adami, Helge G. Roider, Holger Hess-Stumpp, Balázs Győrffy, Jelmar Quist, Anita Grigoriadis, Anette Sommer, Andrew N.J. Tutt, David M. Davies, and John Maher

Supplemental Information

TGF-β1 potentiates γδ T-cell adoptive immunotherapy of cancer

Richard E Beatson, Ana C. Parente-Pereira, Leena Halim, Domenico Cozzetto, Caroline Hull, Lynsey M. Whilding, Olivier Martinez, Chelsea A. Taylor, Jana Obajdin, Kim Ngan Luu Hoang, Benjamin Draper, Ayesha Iqbal, Tom Hardiman, Tomasz Zabinski, Francis Man, Rafael T.M. de Rosales, Jinger Xie, Fred Aswad, Daniela Achkova, Chung-Yang Ricardo Joseph, Sara Ciprut, Antonella Adami, Helge G. Roider, Holger Hess-Stumpp, Balázs Győrffy, Jelmar Quist, Anita Grigoriadis, Anette Sommer, Andrew Tutt, David M. Davies, John Maher

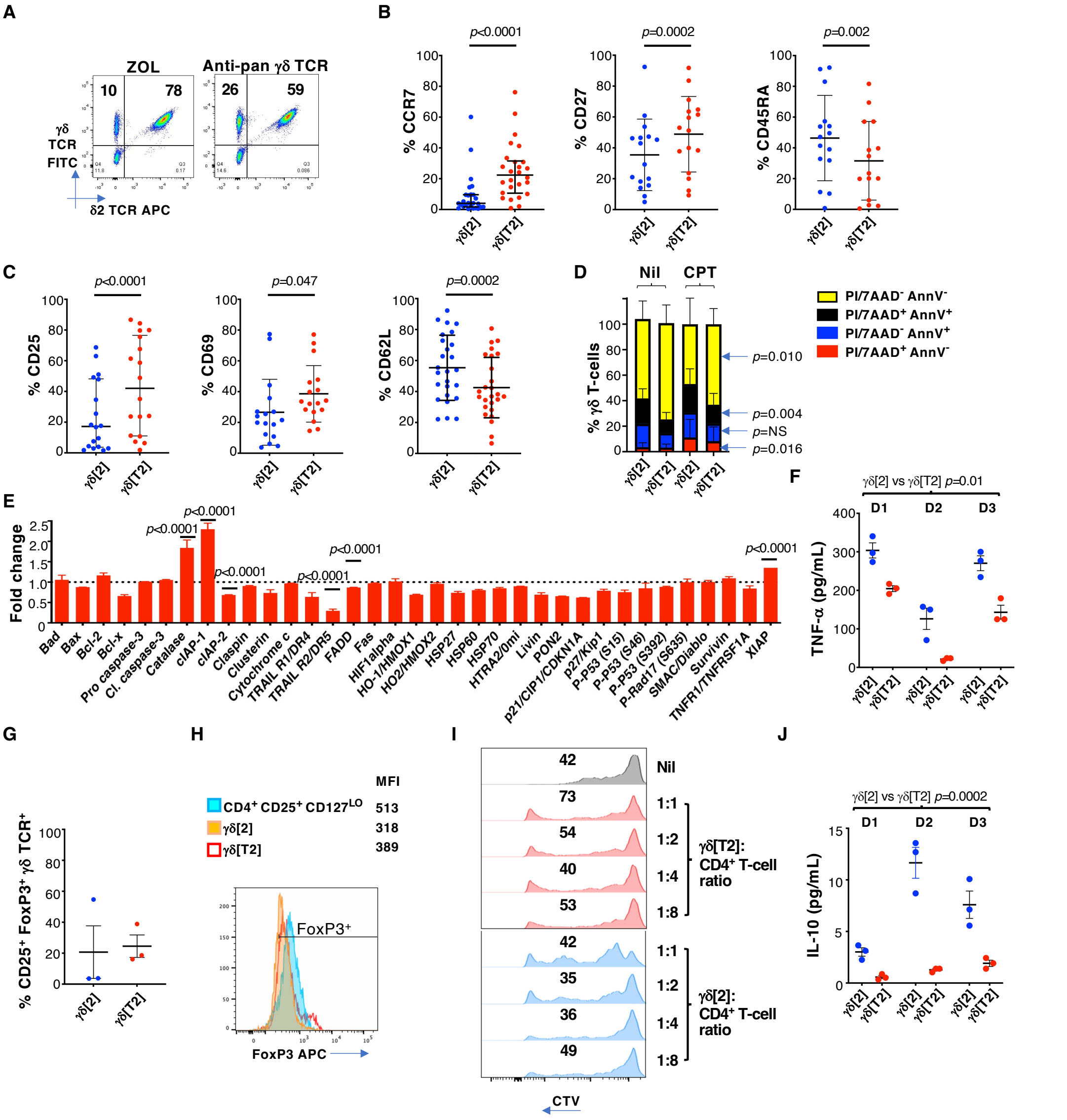

Figure S1. ***In vitro*** **characterization of γδ[T2] cells**

Healthy donor PBMCs were activated with ZOL (n=27) or immobilized anti-γδ TCR antibody (n=15) and then cultured for 14-17 days in SFM containing IL-2 alone (γδ[2] cells) or IL-2 + TGF-β (γδ[T2] cells). (**A**) Representative example of flow cytometric analysis of γδ TCR expression by expanded γδ[2] and γδ[T2] cells after co-staining with fluorochrome-conjugated non-cross reactive pan-γδ TCR and δ2 γδ TCR antibodies. (**B**) Following γδ T-cell expansion, the indicated markers of γδ T-cell differentiation were assessed in γδ[2] and γδ[T2] cells by flow cytometry. (**C**) Expanded cells were also assessed by flow cytometry for the indicated markers of γδ T-cell activation. In B-C, panels show mean +/- SD (CD27, CD45RA, CD69, CD62L) or median +/- interquartile ranges (CCR7, CD25), where data were or were not normally distributed respectively. Accordingly, statistical analysis was performed using a Student's *t*-test or Wilcoxon signed rank test respectively. (**D**) Expanded γδ[2] and γδ[T2] cells were cultured with camptothecin (CPT; 12μM) for 6h and then analyzed for viability by flow cytometry, after incubation with Annexin-V-FITC and 7AAD or PI (mean +/- SD, n=7). Statistical analysis compares camptothecin-treated γδ[2] and γδ[T2] cells using a paired Student's *t*-test or Wilcoxon signed rank test (PI+ AV-). (**E**) Expression of a panel of apoptosis-related proteins was quantified in γδ[2] and γδ[T2] cells using a Proteome Profiler Human apoptosis array kit. Data show pixel density (mean +/- SD, n=2 independent donors). Similar results were obtained in a second independent analysis. (**F**) TNF-α concentration was assessed in supernatant of γδ[2] and γδ[T2] cells from 3 separate donors (D1-D3) following expansion for 14 days. Statistical analysis was by unpaired Student *t*-test. (**G**) FoxP3 and CD25 co-expression in γδ[2] and γδ[T2] cells after 14d expansion (mean +/- SEM, n=3). (**H**) Representative example of FoxP3 expression in γδ[T2] cells (expanded for 14d) and $CD4^+$ $CD25^+$ $CD127^{LO}$ T-regs, enriched by negative selection. Gates were set with an APC-conjugated isotype control antibody. MFI – mean fluorescence intensity. (**I**) Representative suppression assay in γδ[2] and γδ[T2] cells were added to cell trace violet (CTV)-labeled $CD4^+$ cells (depleted of $CD25^+$ $CD127^{LO}$ T-regs) that had been activated with CD3+CD28 beads. Flow cytometry analysis was performed after 96h. (**J**) IL-10 concentration was assessed in supernatant of γδ[2] and γδ[T2] cells from 3 separate donors (D1-D3) following expansion for 14 days. Statistical analysis was by unpaired Student *t*-test. *Related to Figure 1.*

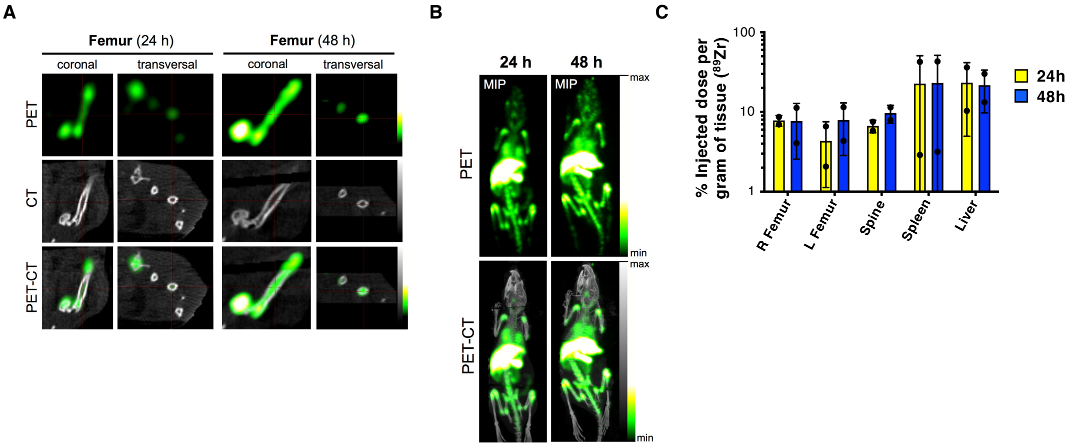

Figure S2. Analysis of trafficking of γδ[T2] cells
[^{89}Zr]Zr(oxinate)$_4$-labeled γδ[T2] T-cells were administered i.v. to NSG mice. Representative images of femur (centered on the medullary cavity; **A**) and whole-body following PET-CT imaging at the specified times (**B**). Proportion of total radioactivity in the indicated organs was determined by gamma counting (**C**). Data were normalized to total activity in each mouse (mean +/- SD n=2; Neg – negative control). *Related to Figure 1.*

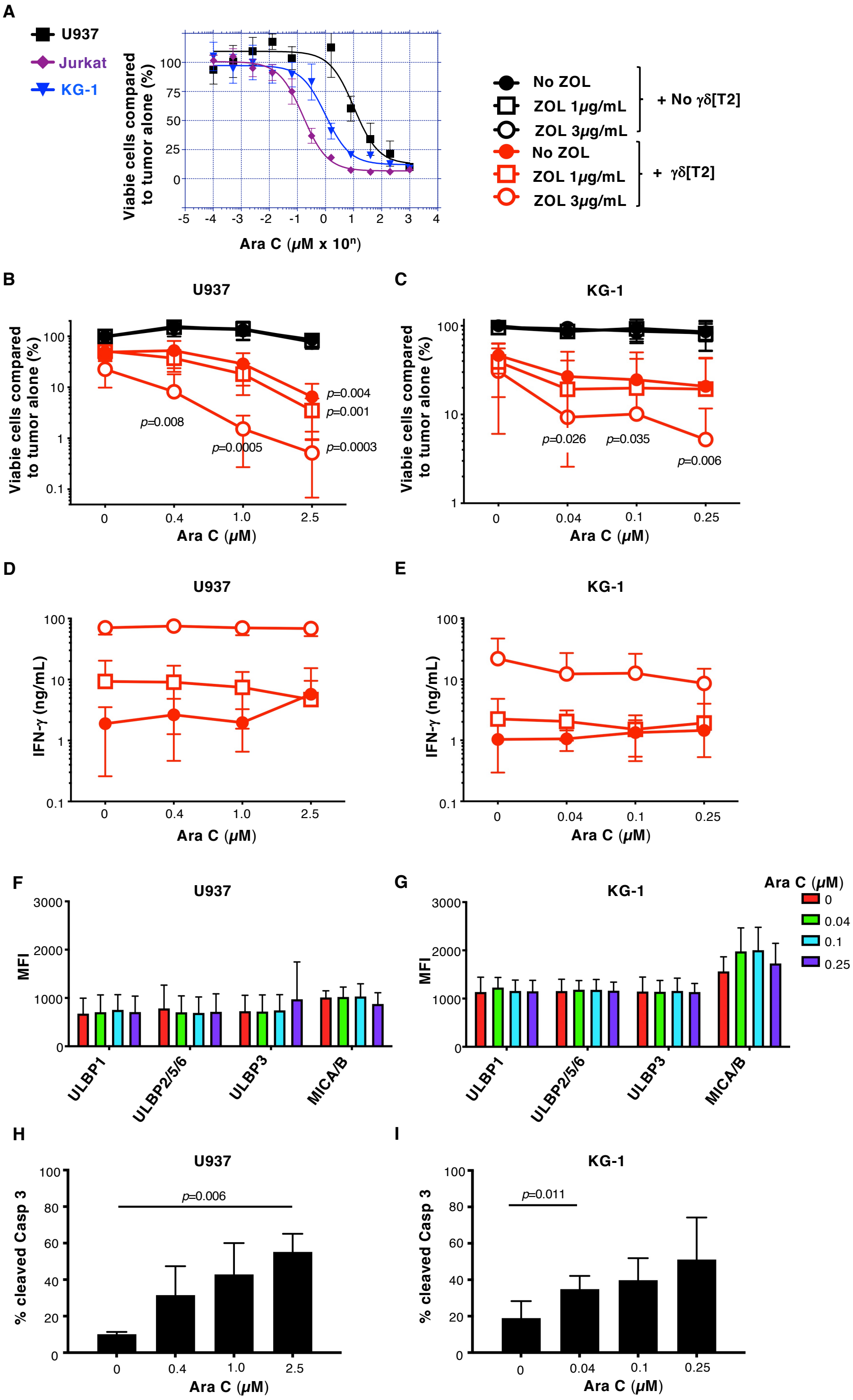

Figure S3. Sensitizing action of Ara C to cytotoxic activity of γδ[T2] cells

(**A**) Firefly luciferase-expressing leukemic cell lines were cultured in Ara C at the specified concentration for 48h. Viability was determined by luciferase assay (mean +/- SD, n=3-5 independent replicates). U937 (**B**) or KG-1 (**C**) cells were sensitized for 24h with the indicated concentrations of Ara C +/- ZOL followed by addition of γδ[T2] cells at a 1:1 E:T ratio. Leukemic cell viability was assessed after a further 24 hours by luciferase assay. Statistical analysis in B-C was performed by two-way ANOVA. *p* values show significance compared to cytotoxic effect of γδ[T2] cells alone, without additional ZOL or Ara-C. IFN-γ content of supernatants derived from U937 (**D**) and KG-1 co-cultures (**E**) was analyzed by ELISA (mean +/- SD, n=3-5 independent replicates). U937 (**F, H**) and KG1 leukemic cells (**G, I**) were treated with the indicated concentration of Ara C for 48 hours and then analyzed by flow cytometry for cleaved Casp(ase) 3 (**F, G**; mean +/- SD, n=3-4 independent replicates) or the indicated NKG2D ligands (**H, I**; mean +/- SD, n=4 independent replicates) . Statistical analysis in H-I was performed using one-way ANOVA. *Related to Figure 3.*

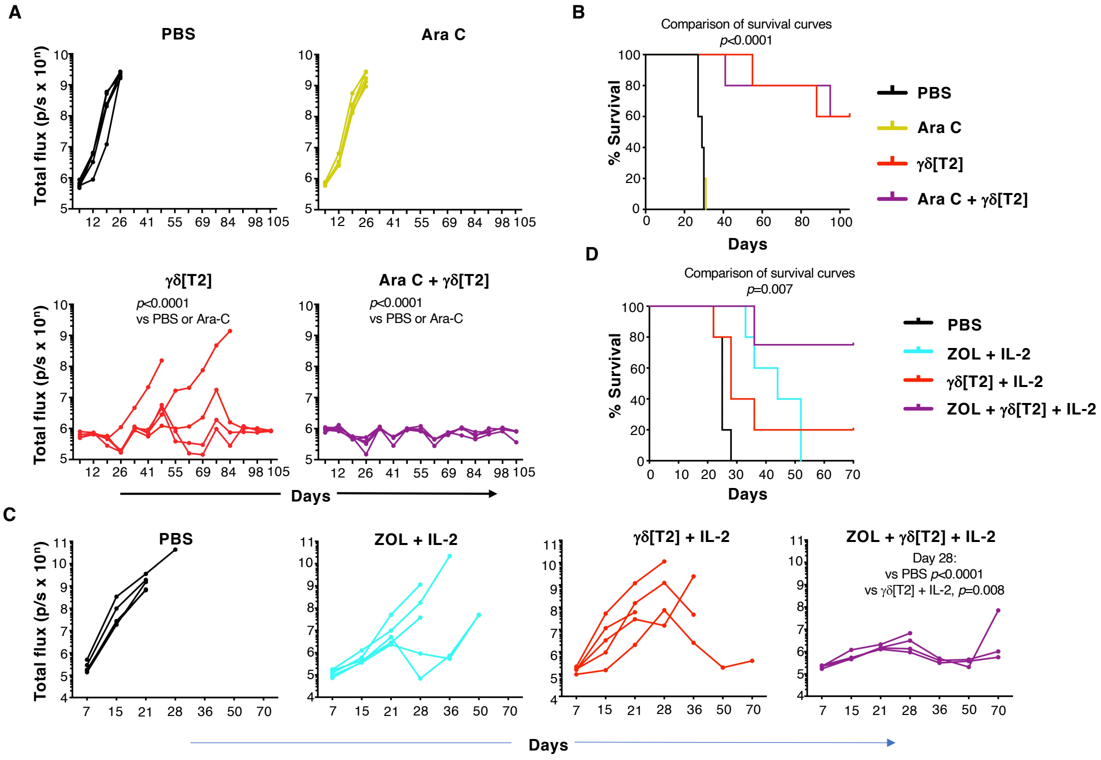

Figure S4. Immunotherapy of leukemia with γδ[T2] cells

(**A**) NSG mice were inoculated i.v. with 1 x 10^5 ffLuc$^+$ Jurkat cells on day 1. Indicated groups of mice received 110mg/kg of Ara C i.v. on day 5 and/or γδ[T2] T-cells (5 x 10^6 cells) i.v. on day 6. Serial BLI emission from individual mice is shown. (**B**) Kaplan Meier survival curve of mice shown in panel A. Two mice in the ZOL + Ara C + γδ[T2] group died of infection and were disease free. (**C**) SCID Beige mice were inoculated i.v. with 1 x 10^6 ffLuc$^+$ U937 cells (day 1). Groups of mice received: (i) ZOL (20µg) i.v. on day 2 and/or (ii) γδ[T2] T-cells (15 x 10^6 cells) i.v. on day 3 and (iii) IL-2 (5000i.u.) twice daily i.v. on days 3-5, making comparison with PBS alone. Serial BLI of individual mice is shown. (**D**) A Kaplan Meier survival curve of mice shown in panel C. All BLI data were analyzed using two-way ANOVA, and survival data were analyzed using the Log-rank (Mantel-Cox) test. *Related to Figure 3.*

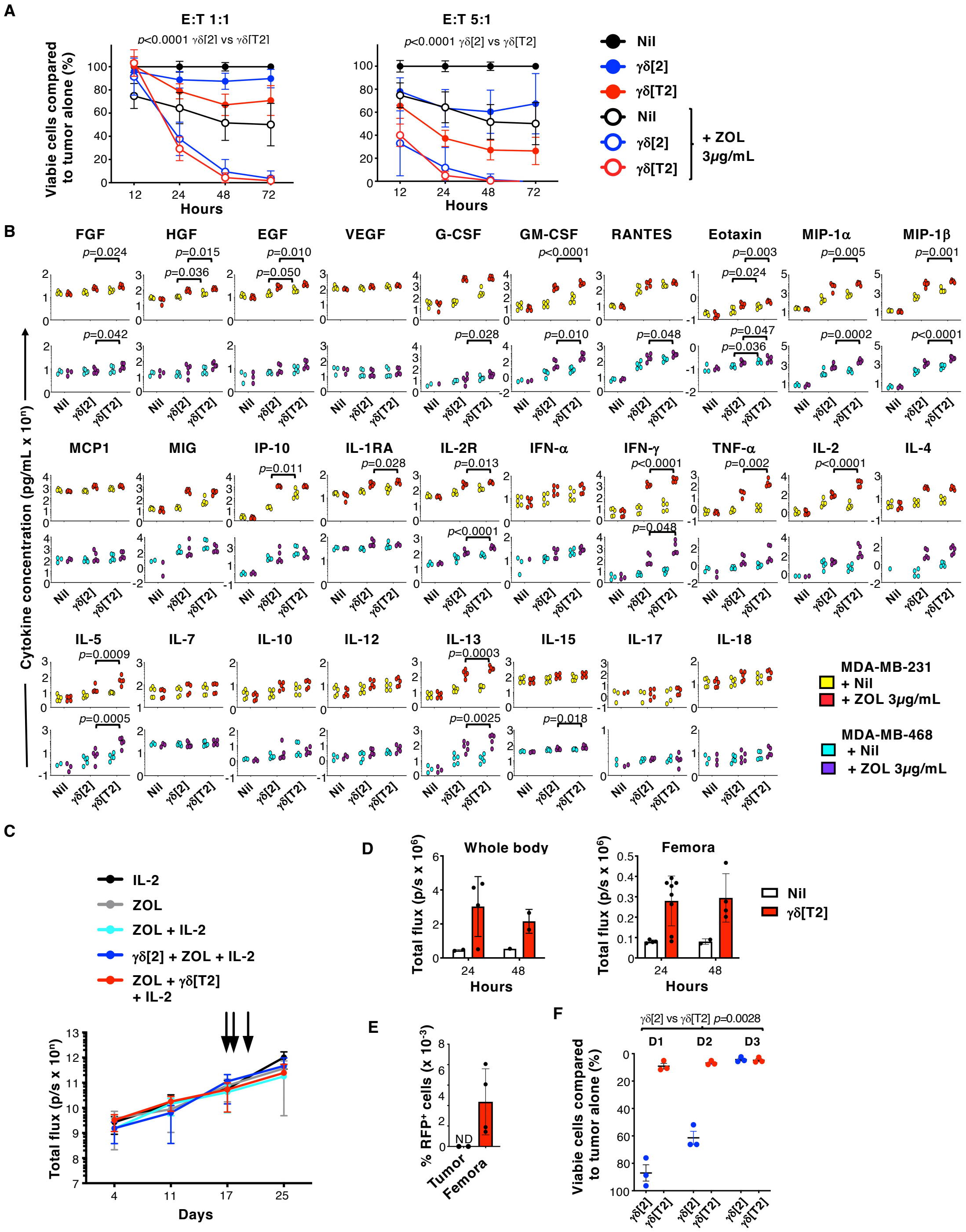

Figure S5. ***In vitro*** **analysis of γδ[2] and γδ[T2] γδ T-cells in solid tumor models**
(**A**) MDA-MB-231 cells ($2x10^4$ cells) were plated were plated on a 96 well electronic microplate. After 24h, cells were pulsed with ZOL (3µg/mL) or media alone. After a further 24h, γδ[2] or γδ[T2] cells were added at the indicated E:T ratio. Dynamic monitoring of adherent tumor viability/ proliferation was performed using an xCELLigence MP impedance analyzer (mean +/- SD n=5). Statistical analysis by two-way ANOVA. (**B**) Supernatants were collected after 24h co-culture of γδ T-cells with MDA-MB-231 and MDA-MB-468 TNBC cell lines at a 5:1 E:T ratio and analyzed for cytokine content using a Luminex array (mean +/- SD n=5). Data are presented on a Log_{10} scale and were analyzed using two-way ANOVA. *p<0.05; **p<0.01; ***p<0.001; ****p<0.0001. (**C**) SCID Beige mice with an orthotopic MDA-MD-231 tumor received ZOL (d17), γδ[2] or γδ[T2] cells (2 x 10^7 cells; d18) and IL-2 (1000 U d20) (all arrowed). Tumor status was imaged by BLI. (**D**) γδ[T2] T-cells were engineered to co-express ffLuc and RFP and 10 million cells injected i.v. into 4 SCID Beige mice with an orthotopic MDA-MB-231 tumor. BLI was performed after 24h and 48h to determine persistence of γδ T-cells in the whole body and in a femoral region of interest. (**E**) After 48h, the % RFP^+ γδ[T2] cells present in tumor (n=2) and bone marrow (n=4 femora) were determined. (**F**) SH-SY5Y neuroblastoma cells were pulsed with ZOL (1µg/mL). After 24h, γδ[2] or γδ[T2] cells (E:T ratio 5:1) from 3 separate donors (D1-D3) were added. Residual tumor cell viability was assessed after a further 24h. Statistical analysis by unpaired Student's t-test. All data are presented as mean +/- SD. NS – not significant. ND – not detected. *Related to Figure 4.*

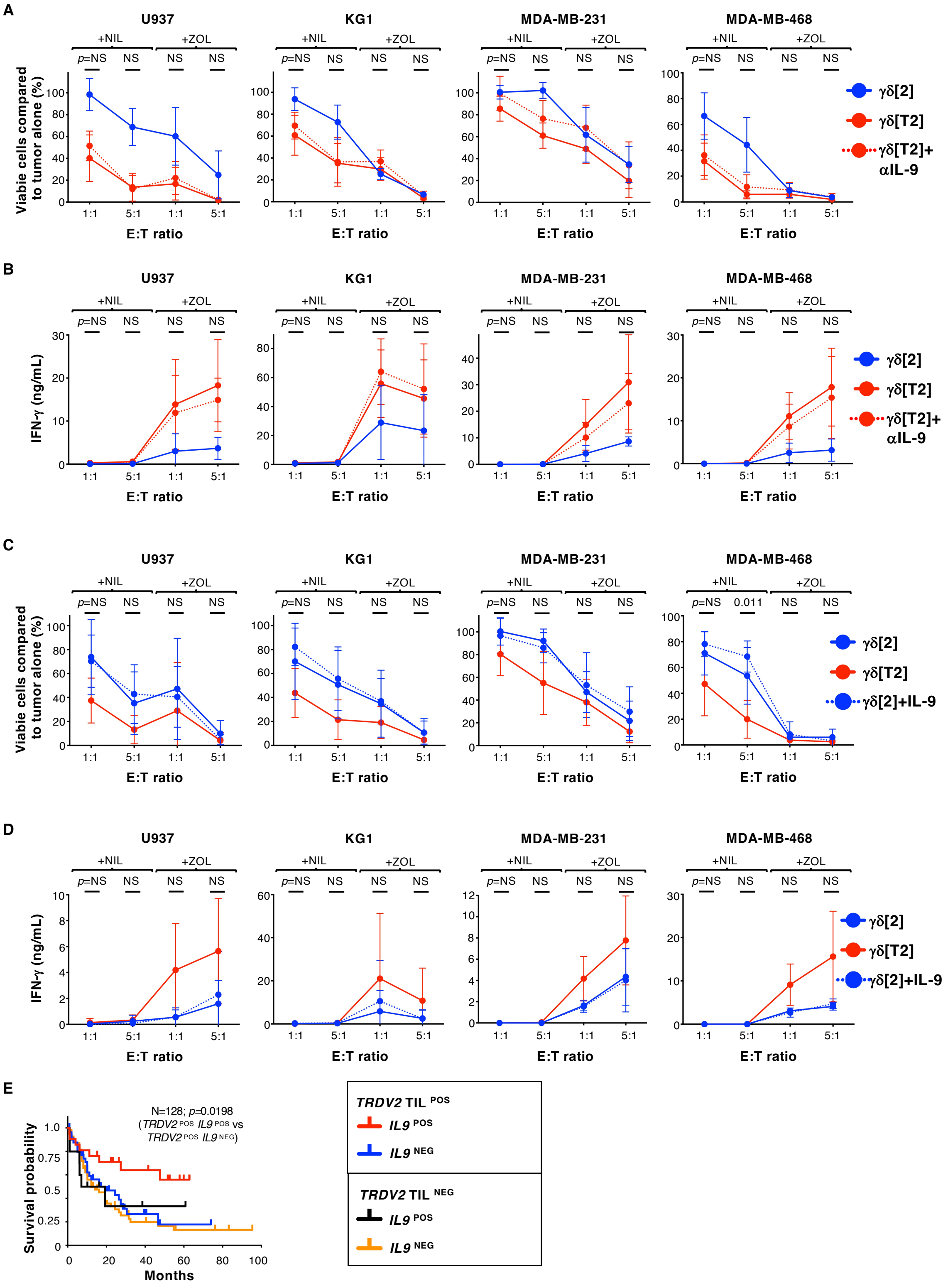

Figure S6. Role of IL-9 in function of γδ[T2] cells

Luciferase-expressing tumor cells were incubated +/- ZOL (3μg/mL) for 24h prior to co-culture for 24h at E:T ratios 1:1 and 5:1 with γδ[2] cells (**A**-**D**), γδ[T2] cells (**A**-**D**), γδ[T2] cells that had been expanded in the presence of αIL-9 blocking antibody (3μg/mL; concentration greater than ND50 for 5ng/mL IL-9; **A**-**B**) or γδ[2] cells that had been expanded in IL-9 (30ng/mL; **C**-**D**). Both IL-9 and αIL-9 blocking antibody were replenished in cultures 3 times per week. Percentage viability was determined by luciferase assay after 24h, making comparison with untreated tumor cells (**A**, **C**). Supernatants collected from these co-cultures after 24h were analyzed for IFN-γ production by ELISA (**B**, **D**). All data show mean +/- SD (n = 9-21 replicates in A, C; 5-14 in B, D) and were analyzed using two-way ANOVA. *p* values shown above each E:T ratio compare γδ[T2] versus γδ[T2]+αIL-9 (A,B) or γδ[2] versus γδ[2]+IL-9 (C,D). (**E**) The impact of *IL9* on survival in AML was determined using Kmplot and Log-rank test analysis for tumors in which *TRDV2* TILs were present or absent (numbers per cohort indicated). Top (POS) and bottom (NEG) thirds are shown for *IL9* in each case. *Related to Figure 6.*

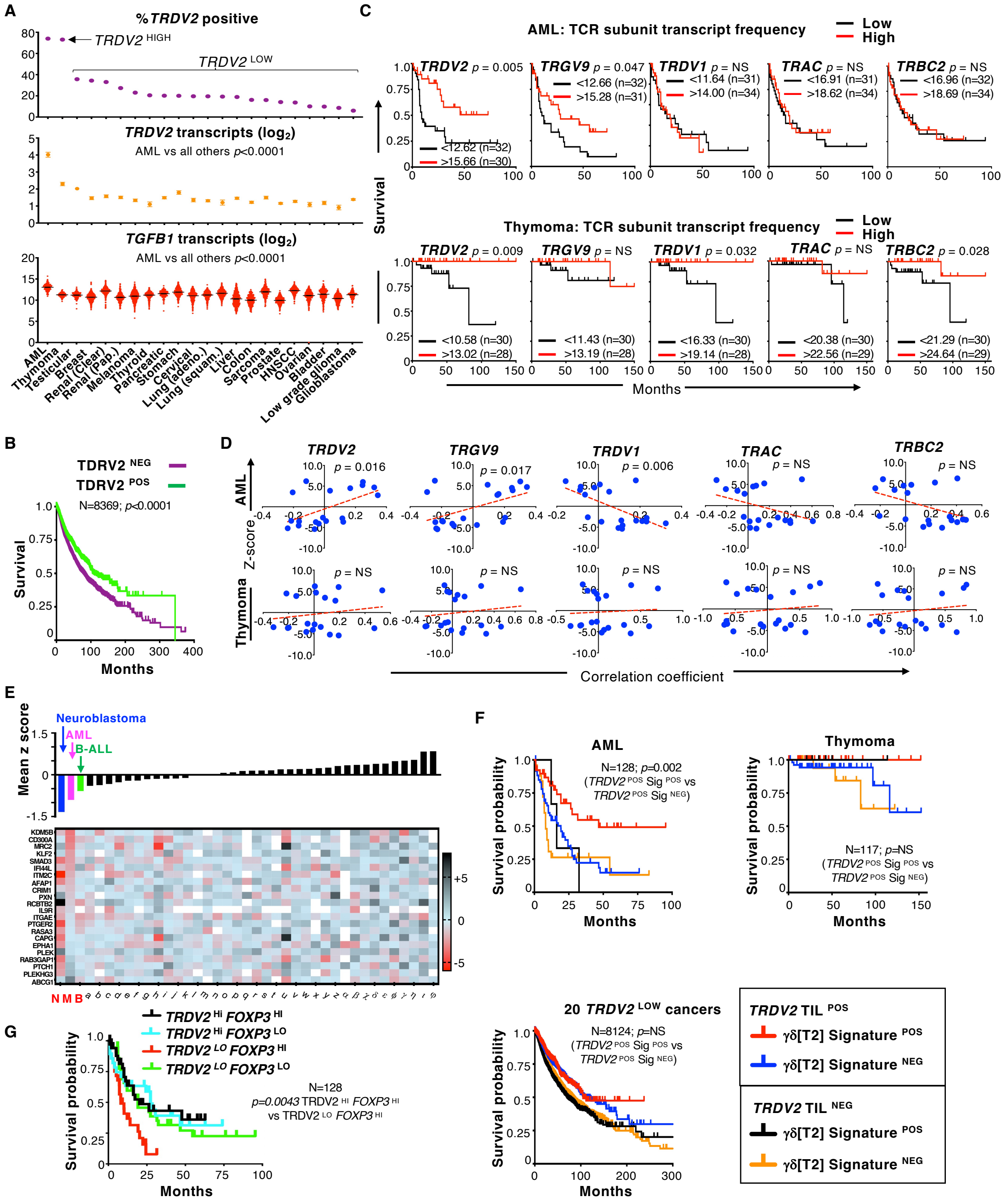

Figure S7. A γδ[T2] gene signature is present in AML and is associated with improved prognosis.
(**A**) Percentage of the indicated 22 cancers (N=8369) in which *TRDV2* transcripts were detected (TCGA; top panel). Transcript quantification is shown in the middle panel. Units are $\log_2$ fragments per kb transcript per million mapped reads upper quartile +1 (mean +/- SEM). Transcripts of *TGFB1* were similarly quantified in these cancers (mean +/- SEM; lower panel). Statistical analysis - one way ANOVA. Adeno – adenocarcinoma; AML, acute myeloid leukemia; clear – clear cell carcinoma; HNSCC, head and neck squamous cell carcinoma; Pap - papillary cell carcinoma; Squam – squamous cell. (**B**) Kaplan Meier curves and Log-rank test analysis showing the association between *TDRV2* expression and survival across 22 cancers listed in A. (**C**) Kaplan Meier curves and Log-rank test analysis showing the association between indicated TCR subunit transcript frequencies and survival in AML and thymoma (TCGA GDC). Patient numbers per cohort are indicated in each plot. (**D**) Correlation coefficient of indicated TCR transcripts and genes that are differentially expressed in γδ[2] and γδ[T2] cells (x axis; AML and thymoma TCGA GDC datasets) was plotted against z-scores of these differentially expressed genes. Statistical analysis was performed using simple linear regression. (**E**) Prediction of clinical outcomes from genomic profiles (PRECOG) analysis querying the association of a transcriptional 24 gene γδ[T2] cell signature in the following cancers: N neuroblastoma; M AML; B B-cell acute lymphoblastic leukaemia; a liver cancer; b mesothelioma; c bladder cancer; d multiple myeloma; e Burkitt lymphoma; f follicular lymphoma; g osteosarcoma; h DLBCL; i adenocarcinoma of lung; j colon cancer; k gastric cancer; l hypopharyngeal cancer; m pancreatic cancer; n small cell carcinoma of lung; o Ewing sarcoma; p oral squamous cell cancers; q medulloblastoma; r squamous cell carcinoma of lung; s meningioma; t kidney cancer; u glioma; v large cell carcinoma of lung; w melanoma; x esophageal cancer; y germ cell tumors; z prostate cancer; α mantle cell lymphoma; β ovarian cancer; χ head and neck cancers; δ breast cancer; ε glioblastoma; ϕ metastatic melanoma; γ astrocytoma; η adrenocortical cancer; ι chronic lymphocytic leukemia; φ liver cancer primary. Mean z scores are shown above the heatmap indicating expression of each differentially expressed gene. (**F**) The impact of γδ[T2] cell signature on survival in AML, thymoma and the remaining 20 *TRDV2* ^{LO} cancer types was determined using Kmplot and Log-rank test analysis for tumors in which *TRDV2* TILs were present or absent (numbers per cohort indicated). Top (POS) and bottom (NEG) thirds are shown for γδ[T2] cell signature in each case. (**G**) The impact of FoxP3 status on survival in AML, ranked for high or low content of *TRDV2* transcripts. Analysis was performed using Kmplot and Log-rank testing. *Related to Figure 6.*
